# Marine-Derived Compounds: A New Horizon in Cancer, Renal, and Metabolic Disease Therapeutics

**DOI:** 10.3390/md23070283

**Published:** 2025-07-09

**Authors:** Jinwei Zhang

**Affiliations:** 1 Institute of Biomedical and Clinical Sciences, Medical School, Faculty of Health and Life Sciences, Hatherly Laboratories, Streatham Campus, University of Exeter, Exeter EX4 4PS, UK; jinweizhang@sioc.ac.cn or j.zhang5@exeter.ac.uk; 2 State Key Laboratory of Chemical Biology, Research Center of Chemical Kinomics, Shanghai Institute of Organic Chemistry, Chinese Academy of Sciences, 345 Lingling Road, Shanghai 200032, China

**Keywords:** marine-derived compounds, cancer therapy, renal disease, autophagy, metabolic-associated fatty liver disease, immune checkpoint inhibitors, kinase inhibitors, PRISMA, pharmacokinetics, structure–activity relationship

## Abstract

Marine-derived compounds represent a rich source of structurally diverse molecules with therapeutic potential for cancer, renal disorders, metabolic-associated fatty liver disease (MAFLD), and atherosclerosis. This review systematically evaluates recent advances, highlighting compounds such as Microcolin H, Benzosceptrin C, S14, HN-001, Equisetin, glycosides (e.g., cucumarioside A_2_-2), ilimaquinone, and Aplidin (plitidepsin). Key mechanisms include autophagy modulation, immune checkpoint inhibition, anti-inflammatory effects, and mitochondrial homeostasis. Novel findings reveal glycosides’ dual role in cytotoxicity and immunomodulation, ilimaquinone’s induction of the DNA damage response, and Aplidin’s disruption of protein synthesis via eEF1A2 binding. Pharmacokinetic challenges and structure–activity relationships are critically analyzed, emphasizing nanodelivery systems and synthetic analog development. This review bridges mechanistic insights with translational potential, offering a cohesive framework for future drug development.

## 1. Introduction

Cancer, metabolic disorders, and renal diseases represent some of the most pressing global health challenges of our time, with significant morbidity and mortality rates worldwide. Despite advances in medical research, current therapeutic strategies for these conditions often fall short due to limitations, such as drug resistance, severe side effects, and incomplete efficacy [1]. For instance, in oncology, while immune checkpoint inhibitors and targeted therapies have revolutionized treatment, a substantial proportion of patients fail to respond or develop resistance over time [2]. Similarly, metabolic-associated fatty liver disease (MAFLD) and acute kidney injury (AKI) lack targeted therapies, with management largely relying on supportive care or lifestyle modifications [3,4]. These unmet clinical needs underscore the urgent demand for novel therapeutic agents that can address the underlying molecular mechanisms of these diseases with greater precision and fewer adverse effects.

Marine-derived compounds have emerged as a promising frontier in drug discovery, offering a rich source of structurally diverse and biologically potent molecules. Unlike terrestrial natural products, marine organisms produce unique secondary metabolites adapted to extreme environments, resulting in chemical scaffolds with unparalleled mechanisms of action. Historically, marine natural products have contributed to the development of groundbreaking drugs, such as the anticancer agent trabectedin and the antiviral drug vidarabine [5,6]. However, the vast potential of marine-derived compounds remains underexplored, particularly in the context of complex multifactorial diseases like cancer, MAFLD, and renal disorders. This review highlights the therapeutic potential of these compounds, focusing on their ability to modulate critical pathways such as autophagy, immune checkpoint regulation, and oxidative stress responses—mechanisms that are often dysregulated in these diseases.

The novelty of this review lies in its systematic integration of marine-derived compound research across oncology, nephrology, and metabolic disease, emphasizing translational strategies that bridge preclinical mechanisms with clinical potential. Unlike prior reviews, this work highlights cross-disease therapeutic frameworks by analyzing conserved molecular targets—such as autophagy (Microcolin H, S14), kinase signaling (nortopsentin and Equisetin), and immune checkpoint regulation (Benzosceptrin C)—that underpin marine compound efficacy [7,8]. For instance, Microcolin H’s induction of autophagic cell death via PITPα/β inhibition [7], exemplifies a novel cancer targeting strategy, while Benzosceptrin C’s lysosomal degradation of PD-L1 [8] offers an innovative approach to overcoming immunotherapy resistance. Additionally, newly added analyses of glycosides (e.g., cucumarioside A_2_-2) [9] and cyclodepsipeptides (e.g., Aplidin) [10] expand the scope to include immune-modulatory mechanisms and protein synthesis disruption, respectively. Compounds like S14 [11] and HN-001 [12] further demonstrate how marine natural products address unmet needs in renal disease (oxidative stress mitigation) and metabolic-associated fatty liver disease (lipotoxicity regulation). By synthesizing recent advances (2020–2025) in pharmacokinetics (e.g., nanodelivery of S14) [13] and combinatorial therapies (e.g., fucoidan-paclitaxel synergy) [14], this review provides a cohesive roadmap for translating marine compounds into clinical applications, addressing challenges like bioavailability and target validation.

## 2. Methodology of Literature Review

**Figure 1:**
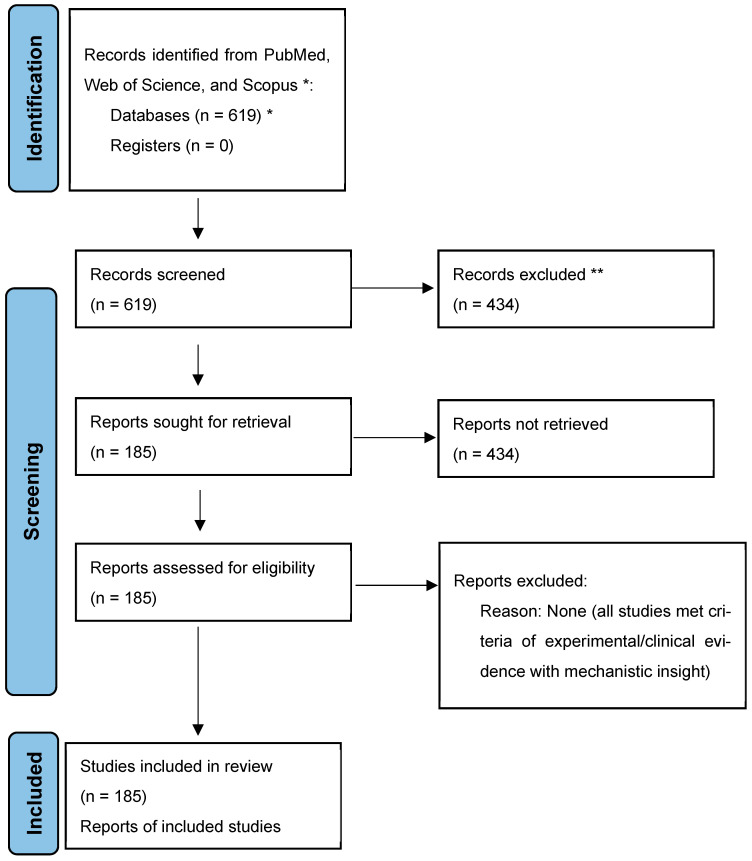
Identification of studies via databases and registers. * A structured search was conducted across PubMed, Web of Science, and Scopus for peer-reviewed publications spanning January 2000 to May 2025. Keyword combinations included “marine-derived compounds”, “natural products”, “cancer therapy”, “renal disease”, “autophagy”, “immunotherapy”, “fatty liver disease”, “STAT3”, and “kinase inhibitors”. ** Reason: Irrelevance to marine compound pharmacology (e.g., terrestrial natural products and non-therapeutic studies). Note: The PRISMA flowchart illustrates the systematic review process, with initial database queries yielding 617 articles. Title/abstract screening excluded 434 studies, and full-text review confirmed 183 articles met the inclusion criteria (experimental/clinical evidence of marine compound activity). This diagram adheres to the PRISMA guidelines (https://pubmed.ncbi.nlm.nih.gov/33782057/, accessed on 29 March 2025) and is licensed under CC BY 4.0.

This study adhered to the Preferred Reporting Items for Systematic Reviews and Meta-Analyses (PRISMA) guidelines to ensure methodological rigor. A structured search was conducted across PubMed, Web of Science, and Scopus for peer-reviewed publications spanning January 2000 to May 2025. Keyword combinations included “marine-derived compounds”, “natural products”, “cancer therapy”, “renal disease”, “autophagy”, “immunotherapy”, “fatty liver disease”, “STAT3”, and “kinase inhibitors”. Initial database queries yielded 619 articles. Title/abstract screening excluded 434 studies, primarily for irrelevance to marine compound pharmacology. Full-text review of the remaining 185 articles excluded studies lacking biological validation or relying solely on computational analysis. The final dataset of 185 articles, selected for their experimental/clinical evidence of marine compound activity and mechanistic clarity, is visualized in the PRISMA flowchart (Figure 1). This systematic approach ensures comprehensive coverage of translational research in marine-derived therapeutics (Table 1).

## 3. Marine-Derived Compounds in Cancer Therapy

### 3.1. Microcolin H

Microcolin H, a marine-derived lipopeptide, functions as a ligand for phosphatidylinositol transfer proteins, specifically targeting the PITPα and PITPβ isoforms.


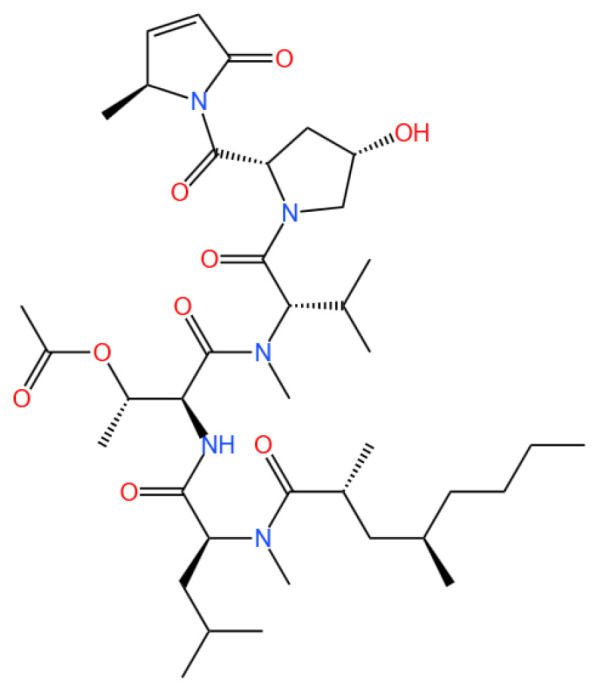


#### 3.1.1. Background and Discovery

Microcolins are a group of compounds that have been isolated from the marine cyanobacterium *Moorea producens*, previously known as *Lyngbya majuscule* [45]. These compounds, including Microcolin A–D and Majusculamide D, were first identified in 1988 and have demonstrated broad antitumor activities against various human cancer cell lines in vitro [45,46,47,48,49,50]. In 2019, additional microcolins, labeled as Microcolin E-M, were discovered and exhibited significant cytotoxic effects, particularly against lung cancer cells [51]. In 2022, Microcolin B (MCB) was identified as a Hippo pathway activator due to its ability to selectively eliminate YAP-dependent cancer cells [52]. The potential of microcolins in cancer treatment is underscored by their ability to induce autophagy, a critical physiological process involved in maintaining cellular homeostasis through the degradation and recycling of cellular components [53].

#### 3.1.2. Mechanism of Action

Autophagy plays a dual role in cancer, where it can either support cancer cell survival or lead to cell death, depending on the context [54]. This duality makes autophagy modulation a promising strategy in anticancer research. It targets phosphatidylinositol transfer protein alpha/beta isoform (PITPα/β), which is implicated in lipid metabolism and membrane trafficking. By binding to PITPα/β, it inhibits cancer cell proliferation and migration, and induces autophagic cell death, as evidenced by increased LC3II conversion and decreased p62 levels.

Microcolin H, a member of this family, has been identified as a novel autophagy inducer with potent antitumor activity. It targets phosphatidylinositol transfer PITPα/β, which are proteins involved in lipid metabolism and membrane trafficking [55]. The expression of PITPα/β is associated with poor prognosis in cancer [56], making them attractive targets for therapeutic intervention.

#### 3.1.3. Experimental Evidence

A Kaplan–Meier survival curve analysis by Yang et al. (2023) demonstrated that reduced expression of PITPα/β is significantly associated with prolonged overall survival and delayed disease progression in gastric cancer patients [7]. Their study then systematically explored the antitumor mechanism of Microcolin H through a combination of chemical proteomics and biological assays [7]. The study achieved large-scale preparation (200 mg grade) of Microcolin H via a multi-step chemical synthesis and validated its activity in both in vitro and in vivo models [7]. In vitro experiments demonstrated that in gastric cancer cell lines (HGC-27, AGS, and MKN-28), Microcolin H inhibited cell proliferation in a dose-dependent manner (CCK-8 assays showed significant inhibition of cell viability at 0.1–0.5 nM (Figure 3a in [7])) and reduced colony formation ability as well as migration capacity (Figure 3b–d in [7]). By contrast, normal gastric mucosal epithelial cells (GES-1) were insensitive to the compound, reflecting its selective toxicity toward tumor cells.

**Figure 2:**
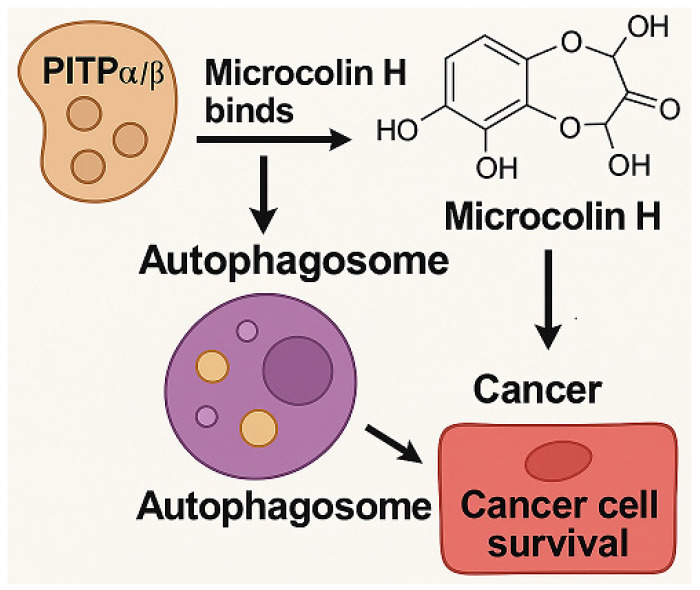
Biochemical mechanism of Microcolin H-mediated autophagy and antitumor activity via PITPα/β targeting. This schematic illustrates the mechanistic action of Microcolin H as a novel autophagy inducer exerting antitumor effects through its direct interaction with phosphatidylinositol transfer proteins PITPα and PITPβ. Upon binding to these lipid transport proteins, Microcolin H promotes autophagosome formation, enhancing autophagic flux. The process leads to the degradation of intracellular cargo, including damaged organelles and oncogenic proteins, thereby contributing to reduced cancer cell survival. The figure includes a depiction of Microcolin H’s chemical structure, the PITPα/β target complex, autophagosome formation, and the resulting suppression of tumor cell viability. This mechanism highlights the therapeutic relevance of targeting lipid transfer proteins in cancer through autophagy modulation.

In vivo efficacy was evaluated using xenograft tumor models in nude mice. Intraperitoneal injection of Microcolin H at doses of 1, 5, and 10 mg/kg exhibited a dose-dependent inhibition of tumor growth, with the 10 mg/kg group achieving a tumor growth inhibition (TGI) rate of 74.2%, which was superior to the positive control paclitaxel (PTX, 8 mg/kg (Figure 7a–c in [7])). A combination with the autophagy inhibitor hydroxychloroquine (HCQ, 50 mg/kg) significantly reversed the antitumor effect of Microcolin H, confirming that its action relies on autophagy activation (Figure 2).

A toxicity assessment revealed no significant changes in mouse body weight during the 11-day treatment period (Figure 7e in [7]), and pathological sections (H&E staining) and biochemical indices of vital organs (heart, liver, spleen, lung, and kidney) showed no obvious abnormalities (Figures 7f and S7b–d in [7]), indicating low toxicity of the compound at the tested doses.

Mechanistic studies and target validation showed that chemical proteomics identified phosphatidylinositol transfer proteins PITPα/β as direct binding targets of Microcolin H (KD = 6.2 μM, MST analysis [7]), through which it induced autophagic cell death. Markers of autophagy, including increased conversion of LC3I to LC3II and decreased p62 levels, were observed (Figure 6a in [7]), and knockout of PITPα/β significantly attenuated Microcolin H-induced autophagy and cytotoxicity (Figure 5b–c in [7]).

The findings by Yang et al. align with previous studies highlighting the dual role of autophagy in cancer, where it can either promote survival or induce cell death [57,58]. Similar to other autophagy inducers like metformin [59], Microcolin H enhances autophagic flux, leading to cancer cell death. However, unlike some autophagy inducers that require combination with other therapies [60], Microcolin H alone demonstrated potent efficacy. This positions it as a promising standalone therapeutic agent, although further studies are needed to explore its full potential and mechanism of action.

#### 3.1.4. Future Perspectives

Future research should focus on elucidating the detailed molecular mechanisms by which Microcolin H modulates autophagy and its potential effects on other cancer types. Investigating the compound’s impact on other diseases associated with PITPα/β, such as neurodegenerative and metabolic disorders, could expand its therapeutic applications. Additionally, exploring combination therapies with existing anticancer agents may enhance its efficacy and broaden its clinical utility.

Microcolin H represents a novel class of autophagy inducers with significant antitumor activity, targeting the previously unexplored PITPα/β proteins. Its ability to induce autophagic cell death in cancer cells, coupled with low toxicity, underscores its potential as a therapeutic agent in oncology. This study provides a foundation for further exploration of Microcolin H and its analogs in cancer therapy and other PITPα/β-related diseases.

### 3.2. Marine Alkaloids: Nortopsentin and Topsentin

Nortopsentin A is a bis(indolyl)imidazole alkaloid.


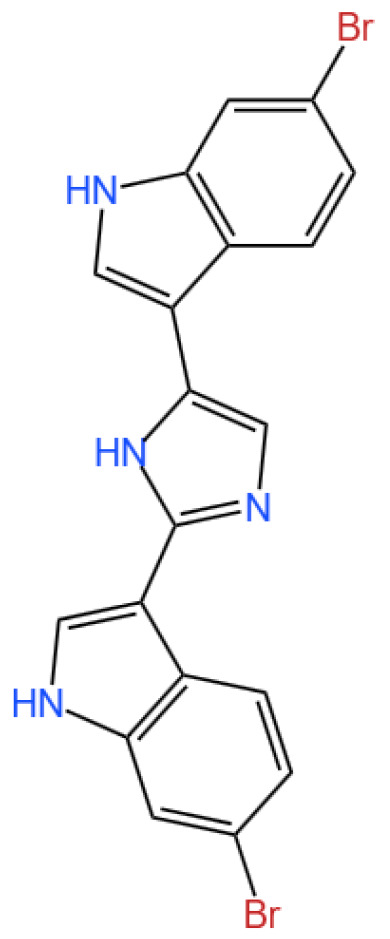


Topsentin is a bis(indolyl) imidazole.


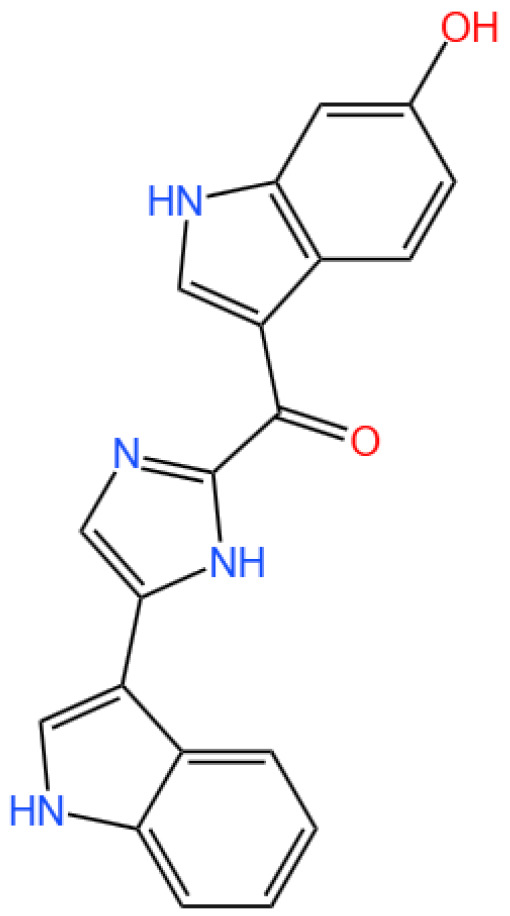


#### 3.2.1. Background and Discovery

Nortopsentin and topsentin, two structurally related bis-indole alkaloids, were initially isolated from marine sponges belonging to the *Topsentia* and *Spongosorites* genera, respectively [61,62]. Their discovery has been pivotal in marine natural product research, as these compounds exhibit unique bis-indole core structures and potent biological activities that distinguish them from terrestrial alkaloids. Early studies highlighted their remarkable antiproliferative effects against cancer cells and their ability to modulate critical signaling pathways involved in tumor progression, thus garnering significant attention for their therapeutic potential [15]. Since their isolation, the natural lead structures have served as foundational scaffolds for synthetic modification, enabling researchers to explore analogs with enhanced activity, selectivity, and pharmacological properties [16,63].

#### 3.2.2. Mechanism of Action

The antitumor mechanisms of nortopsentin and topsentin are rooted in their ability to target multiple cellular pathways. Nortopsentin and its synthetic derivatives primarily act as inhibitors of casein kinase 1 (CK1) and glycogen synthase kinase-3β (GSK-3β), two serine/threonine kinases that play central roles in regulating the Wnt/β-catenin signaling pathway, which is often hyperactivated in colorectal and pancreatic cancers [9,64,65]. By disrupting this pathway, these compounds induce cell cycle arrest and promote apoptosis in cancer cells. Specifically, modifications to the indole scaffold of nortopsentin—such as substitutions with 1,3,4-oxadiazole or thiadiazole moieties—have been shown to enhance binding affinity to CK1 and GSK-3β, reducing off-target effects and improving selectivity [16,66]. Similarly, topsentin analogs target kinases like CDK1 and GSK-3β, as well as tubulin, to disrupt mitosis and cell proliferation [65,67]. These multi-target mechanisms underscore the structural versatility of these alkaloids and their potential to address complex oncogenic pathways.

#### 3.2.3. Experimental Evidence

Preclinical investigations have provided robust evidence of the therapeutic potential of nortopsentin and topsentin. For instance, synthetic analogs of nortopsentin have demonstrated submicromolar to micromolar EC_50_ values against pancreatic ductal adenocarcinoma (PDAC) and colorectal cancer cells, including variants resistant to conventional therapies like gemcitabine [16,17]. Flow cytometric analyses have confirmed that these analogs induce cell cycle arrest at the G1 or G2-M phase by inhibiting cyclin-dependent kinases (CDK1 and CDK6), while molecular docking studies have visualized their direct binding to kinase active sites [16,17]. In colorectal cancer models, analog 4i—a nortopsentin derivative—exhibited particularly potent activity, downregulating CDK2, CDK4, and CDK6 expression and suppressing CDK6 enzymatic activity to induce G1-phase arrest [17]. Theoretical studies further validate the stability and drug-like properties of these analogs, with quantum chemical analyses confirming compliance with Lipinski’s rule of five and favorable energy gaps (ΔE) for biological activity [17]. Additionally, topsentin derivatives have shown efficacy against breast cancer cells, inhibited bacterial biofilm formation, and demonstrated photoprotective effects in UV-irradiated keratinocytes, highlighting their multifaceted biological activity [18,63,67,68,69].

#### 3.2.4. Future Perspectives

The ongoing research on nortopsentin and topsentin points to several promising directions for drug development. Structural optimization of the bis-indole core—such as introducing heterocyclic substitutions or halogenated groups—aims to further enhance kinase inhibition and overcome drug resistance in cancer cells [67,70]. Combination therapies represent another avenue, as nortopsentin analogs have shown synergistic effects with CHK1 inhibitors in sensitizing drug-resistant cancer stem cells, which warrants exploration in preclinical and clinical settings [71]. Furthermore, the discovery of topsentin’s potential as an adenosine A2A receptor antagonist and anti-inflammatory agent expands its therapeutic scope beyond oncology [62]. Advances in total synthesis, such as the development of efficient pinacol-like rearrangement reactions, have enabled large-scale production of these analogs, facilitating their preclinical evaluation and paving the way for translational research [72]. Collectively, these efforts highlight the significant potential of nortopsentin and topsentin as innovative scaffolds for the development of novel anticancer and antimicrobial agents, exemplifying the value of marine natural products in addressing unmet clinical needs.

### 3.3. Bryostatin

Bryostatin 1 is a macrocyclic lactone.


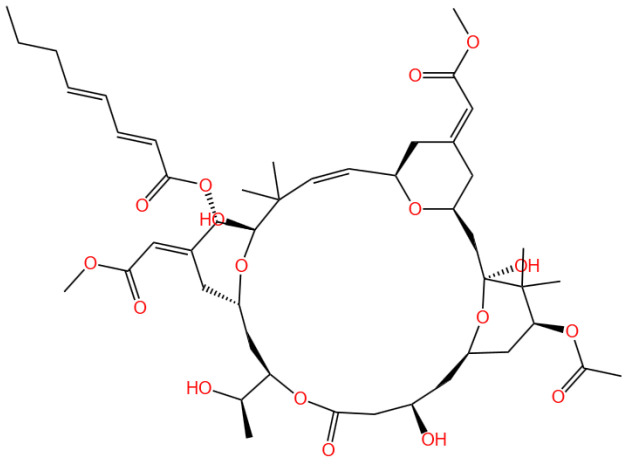


#### 3.3.1. Background and Discovery

Bryostatins are a family of marine-derived macrolides initially isolated from the bryozoan Bugula neritina [73], garnering attention for their potent biological activities and therapeutic potential. The most studied analog, bryostatin-1, has undergone over 40 clinical trials, with the U.S. FDA granting orphan drug status for indications including esophageal cancer (in combination with paclitaxel) and fragile X syndrome [74]. Their discovery stemmed from investigations into marine natural products as sources of novel therapeutics, and synthetic biology advancements have enabled total syntheses of bryostatins 1, 3, 7, 9, and analogs [75,76], addressing challenges of natural availability. These compounds stand out as protein kinase C (PKC) modulators, distinct from phorbol esters, as they exhibit anticancer properties rather than tumor-promoting effects [74], positioning them as promising candidates for multi-disease intervention.

#### 3.3.2. Mechanism of Action

Bryostatin exerts its effects by binding to and activating specific PKC isozymes, including α, β, δ, and ε [19,20,21], thereby regulating downstream signaling pathways. Unlike phorbol esters, bryostatin induces a unique conformational change in PKC, promoting its translocation to the cell membrane and sustained activation [60]. This mechanism underlies its diverse biological effects: in cancer, it enhances CD72 surface antigen density on B-cell malignancies to improve CAR T cell targeting [77], while in inflammation, it suppresses TRPM8 mRNA and induces TRPV1 mRNA in lung epithelial cells, reducing pro-inflammatory responses [19]. In vascular health, bryostatin-1 restores shear-stress-induced eNOS phosphorylation in endothelial cells, improving vascular function [22]. Additionally, it modulates T cell exhaustion by upregulating MAP Kinase 11 in exhausted CD8+ T cells [20] and shifts microglia/macrophages toward a regenerative phenotype in the central nervous system (CNS), attenuating neuroinflammation [21].

#### 3.3.3. Experimental Evidence

Preclinical and clinical studies have validated bryostatin’s efficacy across multiple disease areas. In oncology, bryostatin-1 enhances elimination of antigen-low B-cell malignancies by increasing target antigen density [77] and activates pro-apoptotic ERK signaling in acute erythroleukemia via TGF-β pathway inhibition [78]. For neurodegenerative disorders, it improves cognitive function in Alzheimer’s disease models by promoting synaptogenesis and reducing amyloid/tau pathology [79,80], while in multiple sclerosis, it promotes remyelination by reprogramming microglia [21]. In HIV research, bryostatin-1 reactivates latent virus in CD4+ T cells [23,81], though its effect is limited in macrophages [23]; combined with other latency-reversing agents, it shows synergistic potential [82]. Additionally, bryostatin-1 protects against intestinal ischemia-reperfusion injury by activating Nrf2/HO-1 signaling [83] and improves vascular function in aged models by restoring endothelial autophagy [22].

#### 3.3.4. Future Perspectives

Ongoing research aims to overcome translational barriers, such as bryostatin’s complex synthesis and short half-life. Advances in total synthesis (e.g., scalable production of bryostatin-1) [75,84] and nanoformulations (e.g., exosome/ferritin encapsulation) [85,86] enhance bioavailability and CNS penetration. Machine learning-driven screening seeks to design PKC ligands with reduced inflammatory potential [87], while combinatorial therapies (e.g., bryostatin-1 with checkpoint inhibitors or histone deacetylase inhibitors) show promise in preclinical models [82,88]. Targeted delivery systems, like CD4+ T cell-targeted nanoparticles for HIV [89], and investigations into isoform-specific PKC modulation (e.g., PKCε for neurodegeneration) [90] may refine therapeutic indices. Clinical trials exploring bryostatin-1 in autism spectrum disorder and amyotrophic lateral sclerosis (ALS) highlight its expanding therapeutic horizon [90,91], though long-term safety and efficacy in diverse patient populations require further validation.

### 3.4. Benzosceptrin C

Benzosceptrin C is a pyrrole-2-aminoimidazole alkaloid.


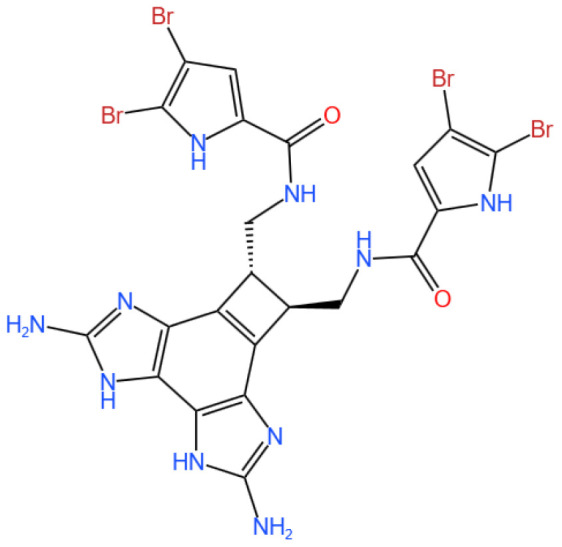


#### 3.4.1. Background and Discovery

**Figure 3:**
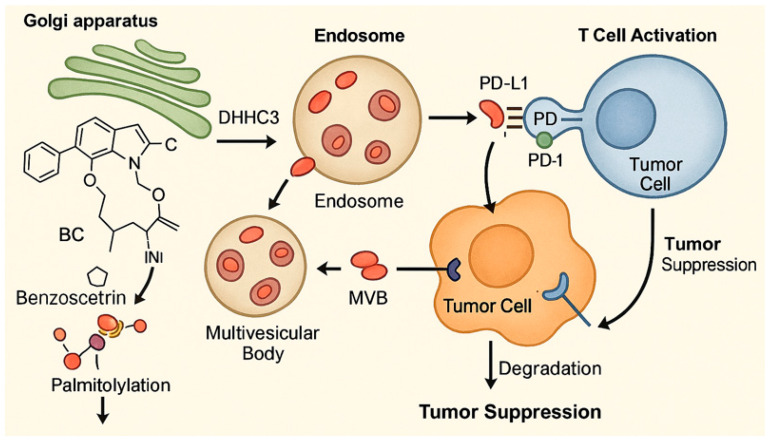
Mechanism of action of Benzosceptrin C in PD-L1 degradation and T cell activation. This schematic illustrates the cellular pathway by which Benzosceptrin C (BC) induces the degradation of PD-L1, leading to enhanced T cell cytotoxicity and tumor suppression. BC interferes with the palmitoylation process of PD-L1 by inhibiting DHHC3 activity within the Golgi apparatus. This inhibition results in decreased stability of PD-L1, leading to its ubiquitination and subsequent degradation via the lysosomal pathway. The figure also highlights the role of endosomes and multivesicular bodies (MVBs) in this process. The activation of T cells is depicted through their interaction with tumor cells, emphasizing the therapeutic potential of combining BC with anti-CTLA4 to suppress tumor growth.

Benzosceptrin C (BC) is a natural marine-derived pyrrole-2-aminoimidazole alkaloid first isolated from the New Caledonian sponge *Agelas dendromorpha* [92], with subsequent isolation from Agelas oroides near the Israeli Mediterranean coast [93] and Agelas sceptrum [94]. Its discovery involved both natural product extraction and synthetic exploration: studies using cell-free enzyme preparations from marine sponges achieved de novo synthesis of BC using labeled oroidin [94], while chemical synthesis via quinone-guanidine reactions provided access to its bis-2-aminobenzimidazole moiety [95]. Biochemically, BC induces lysosomal degradation of programmed cell death ligand-1 (PD-L1) to enhance T cell-mediated antitumor immunity by targeting DHHC3 (Figure 3) [8] and inhibits growth and biofilm formation in *Pseudomonas aeruginosa* [93]. Simplified benzazole fragments derived from BC’s structural motifs have been developed as necroptosis inhibitors targeting receptor-interacting protein kinase-1 [96], underscoring its role in both natural product chemistry and therapeutic research.

#### 3.4.2. Immunotherapy Context

Cancer immunotherapy has revolutionized oncology, with immune checkpoint inhibitors targeting the programmed cell death-1 (PD-1)/PD-L1 axis being among the most successful approaches [97]. However, their clinical efficacy is limited by immune resistance mechanisms and adverse effects [98]. PD-L1, a key immune checkpoint molecule, facilitates tumor immune evasion by binding to PD-1 on cytotoxic T cells, suppressing their activity [99]. Post-translational modifications, such as palmitoylation, stabilize PD-L1 on the tumor cell membrane, protecting it from degradation [24,100]. Targeting these modifications offers a novel avenue to disrupt PD-L1-mediated immune suppression [100]. However, limitations such as immune resistance and adverse effects persist.

#### 3.4.3. In Vivo and In Vitro Results, and Mechanism of Benzosceptrin C

A recent study by Wang et al. (2024) explores the potential of Benzosceptrin C (BC), a natural compound isolated from marine sources, to induce PD-L1 degradation and enhance antitumor immunity by targeting DHHC3, the enzyme responsible for PD-L1 palmitoylation (Figure 3) [8]. The drug effect on PD-L1 expression was evaluated using colorectal cancer (CRC) cell lines (RKO and HCT116) and a murine MC38 tumor model. In vitro experiments applied BC at dosages of 10 μM and 20 μM, with DMSO as the vehicle control and a known PD-L1 inhibitor (e.g., CMPD1) as the positive control to assess PD-L1 degradation. Other techniques included Western blotting, immunofluorescence, flow cytometry, and molecular docking to identify and confirm BC’s interaction with DHHC3.

For in vivo studies, mice bearing MC38 tumors were treated with BC via intraperitoneal injection at 5 mg/kg and 10 mg/kg, while the positive control group received an isotype-matched IgG antibody, and the vehicle control group received saline. Functional assays assessed T cell-mediated cytotoxicity, and combination therapies with anti-CTLA4 (administered at 200 μg/mouse twice weekly) were tested in vivo to evaluate synergistic effects. Key findings revealed that BC significantly reduced PD-L1 levels in CRC cells by inhibiting its palmitoylation via DHHC3, preventing PD-L1 recycling to the cell membrane and redirecting it to lysosomal degradation. In vivo, BC treatment at 10 mg/kg suppressed tumor growth by 45% compared to the vehicle control, increased CD8+ T cell infiltration by 2.3-fold, and reduced immunosuppressive Tregs and myeloid-derived suppressor cells (MDSCs). Combination therapy with anti-CTLA4 further enhanced these effects, with a 60% tumor growth inhibition rate. Importantly, BC exhibited minimal systemic toxicity in treated mice, as indicated by normal body weight changes and organ histology across all dosage groups.

BC’s dual action on PD-L1 degradation and immune activation positions it as a promising alternative to monoclonal antibodies targeting PD-1/PD-L1. Compared to existing small molecules, BC’s ability to disrupt post-translational modifications provides a unique mechanism of action. However, its efficacy in other tumor types (e.g., lung or breast cancer) and its pharmacokinetics—including half-life and bioavailability—require further exploration using different dosage regimens. Studies like Yao et al. (2019), which used PD-L1 palmitoylation inhibitors at 5 μM as positive controls, highlight the need for direct comparisons of efficacy and safety profiles across compounds [24].

#### 3.4.4. Future Research Directions

The findings from Wang et al.’s study open new avenues for the development of marine-derived compounds as potential immunotherapeutic agents. Future research should aim to broaden the application of BC’s efficacy to a wider range of cancer types, ensuring its versatility in cancer treatment. Additionally, it is important to investigate the long-term safety and pharmacokinetics of BC to better understand its effects and duration in the body. Efforts should also be made to optimize BC’s molecular structure to improve its binding affinity and stability, which could enhance its therapeutic effectiveness. Furthermore, exploring BC’s potential in combination with other immune checkpoint inhibitors or conventional therapies could provide synergistic effects, improving treatment outcomes for patients.

BC represents a novel class of immune checkpoint modulators, targeting PD-L1 palmitoylation to enhance antitumor immunity. Its unique mechanism of action, coupled with its efficacy in combination therapies, underscores its potential for clinical application in cancer treatment.

### 3.5. Glycosides

Frondoside A is a triterpenoid glycoside.


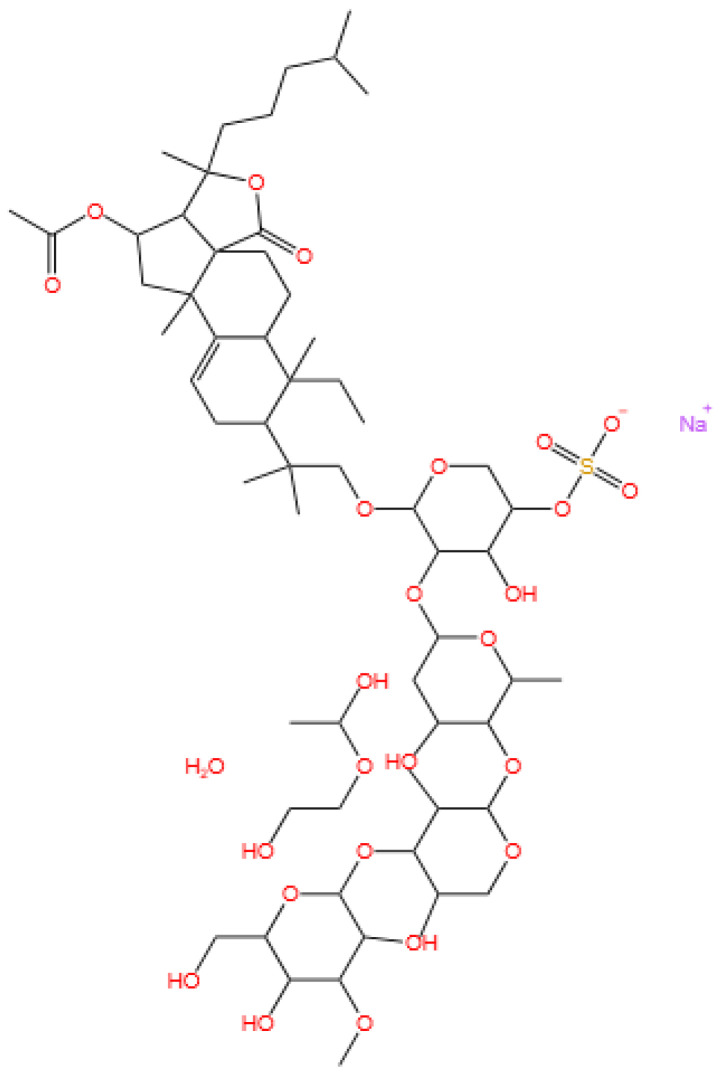


Cucumarioside A_2_-2 is a triterpene glycoside.


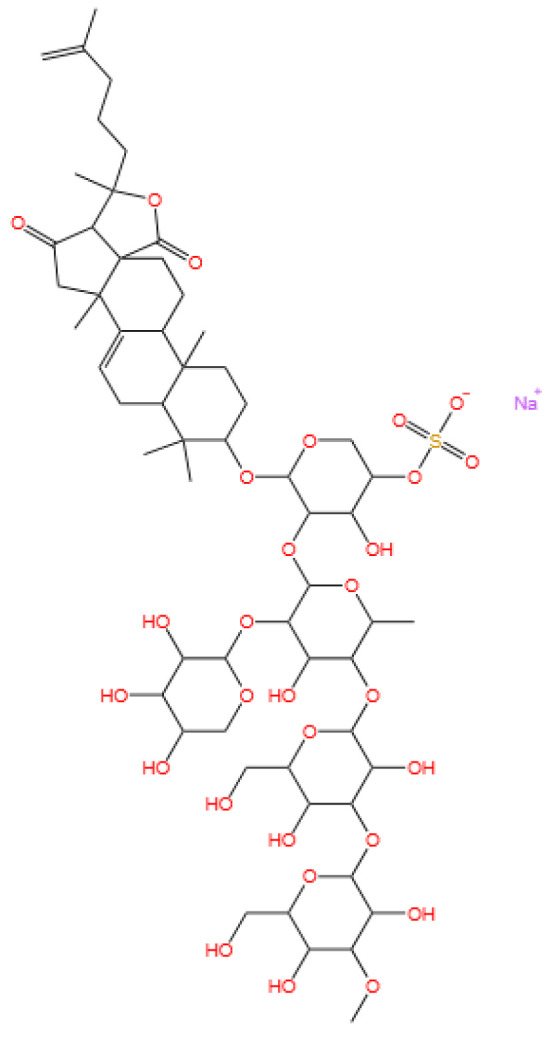


#### 3.5.1. Background and Discovery

Marine-derived glycosides, particularly triterpene glycosides and steroidal glycosides, represent a diverse class of natural products with unique structural complexity and therapeutic potential. These compounds are predominantly isolated from sea cucumbers (e.g., *Cucumaria conicospermium* and *Colochirus quadrangularis*), starfish, and marine bacteria, where they often serve as defensive metabolites or membrane-active agents [101,102]. For example, five new triterpene pentaosides (conicospermiumosides A3-1 to A7-2) were recently isolated from the Far Eastern sea cucumber *C. conicospermium*, featuring sulfated carbohydrate chains and diverse aglycone structures [101]. Similarly, the sea star *Patiria pectinifera* yields pectiniferosides A-J, polyhydroxy steroidal glycosides with sulfated/methylated monosaccharides, highlighting the structural diversity of glycosides in echinoderms [103].

Marine bacteria also contribute to glycoside discovery. For instance, the deep-sea bacterium *Bacillus licheniformis* KDM612 biotransforms seriniquinone into a new glycoside with enhanced solubility, demonstrating microbial potential in glycoside biosynthesis [104]. Additionally, soft corals like *Lemnalia bournei* produce diterpene glycosides (lemnaboursides H-I) with antibacterial activity, expanding the ecological roles of marine glycosides [105]. Additionally, the triterpene glycoside cucumarioside A_2_-2, isolated from the Far Eastern sea cucumber *Cucumaria japonica*, has emerged as a promising anticancer agent. This compound belongs to a class of holostane-type triterpene glycosides characterized by a sulfated carbohydrate chain and a unique aglycone structure, distinguishing it from other sea cucumber-derived glycosides [25,26].

#### 3.5.2. Mechanism of Action

Marine glycosides exert their biological effects through multiple mechanisms, often targeting cell membranes, signaling pathways, or metabolic processes. Triterpene glycosides from sea cucumbers, such as frondoside A, induce apoptosis in bladder cancer cells by activating caspase-8 and caspase-3 while also modulating sphingomyelinase activity to promote ceramide production [106,107]. Compounds like coloquadranoside A, isolated from *Colochirus quadrangularis*, exhibit cytotoxicity by disrupting membrane integrity and inhibiting tumor cell proliferation, with IC_50_ values as low as 0.46 μM against human tumor cell lines [102].

Glycosides may also target specific proteins to alter cellular function. The seriniquinone glycoside synthesized by *B. licheniformis* KDM612 maintains melanoma-selective cytotoxicity by targeting dermcidin, a protein associated with cancer cell drug resistance, while its glycosylation improves solubility for therapeutic application [104]. In breast cancer cells, conicospermiumoside A7-1 inhibits colony formation and migration by interfering with membrane lipid organization, particularly in triple-negative breast cancer (TNBC) cell lines [101]. Cucumarioside A_2_-2 exerts the dual mechanisms of anticancer and immunomodulation. In prostate cancer cells, it induces apoptosis by activating the mitochondrial pathway, evidenced by increased cytochrome c release and caspase-3 activation, while also inhibiting androgen receptor signaling to suppress cell proliferation [26]. Concurrently, the compound activates macrophages via toll-like receptor 4 (TLR4)/NF-κB signaling, promoting the secretion of pro-inflammatory cytokines (e.g., TNF-α and IL-6) and polarizing macrophages toward the M1 phenotype, which enhances antitumor immunity [25,27]. This dual activity positions cucumarioside A_2_-2 as a potential immunotherapeutic agent, bridging direct cytotoxicity with immune system activation.

#### 3.5.3. Experimental Evidence

Preclinical studies have validated the bioactivity of marine glycosides across various disease models. In vitro, conicospermiumosides A3-3 and A7-1 show potent cytotoxicity against MDA-MB-231 TNBC cells (IC_50_ < 1 μM) with minimal toxicity to normal cells and inhibit tumor cell migration by disrupting actin cytoskeleton dynamics [101]. Frondoside A demonstrates dose-dependent inhibition of bladder cancer cell viability (UM-UC-3 cells, IC_50_ = 2.5 μM), surpassing the chemotherapeutic agent epirubicin (IC_50_ = 10 μM), and synergizes with CpG oligodeoxynucleotides to suppress tumor growth in nude mice without significant systemic toxicity [106].

Marine glycosides also exhibit promise in combination therapies. Quadrangularisosides from *C. quadrangularis* enhance the anticancer effect of radioactive irradiation on HT-29 colorectal cancer cells, reducing colony formation by 30% when combined with 1 Gy radiation [108]. Similarly, cladolosides from *Cladolabes schmeltzii* show synergistic inhibition of HT-29 cell colonies when paired with radiation, with cladoloside P2 increasing radiation efficacy by 70% [109,110].

Pharmacokinetic studies highlight challenges for clinical translation. While frondoside A demonstrates antitumor activity in vivo, its short half-life and poor bioavailability necessitate nanodelivery systems, such as liposome encapsulation, to improve stability and targeting [106]. The seriniquinone glycoside shows 50-fold higher solubility in DMSO than its aglycone, addressing a key limitation for drug formulation [104]. Preclinical studies on cucumarioside A_2_-2 have demonstrated significant anticancer efficacy. In vitro, the compound inhibits the growth of human prostate cancer cells (PC-3 and LNCaP) with IC_50_ values of 1.2–2.8 μM, surpassing the efficacy of conventional anticancer drugs like docetaxel in specific models [26]. In mouse models, cucumarioside A_2_-2 (5 mg/kg i.p.) reduces tumor volume in prostate cancer xenografts by 45% within 14 days, accompanied by increased infiltration of M1 macrophages and CD8+ T cells [25].

Immunomodulatory experiments show that cucumarioside A_2_-2 activates mouse splenic macrophages, enhancing their phagocytic activity and cytokine production. This effect is comparable to lipopolysaccharide (LPS) but with reduced systemic inflammatory risk, as evidenced by lower serum IL-1β levels in treated mice [27]. The compound also synergizes with immune checkpoint inhibitors, increasing the cytotoxicity of activated T cells against cancer cells in co-culture models [25].

#### 3.5.4. Future Perspectives

Future research on marine glycosides should focus on structural optimization and translational strategies. For instance, modifying sulfate group positions in triterpene glycosides (e.g., tetrasulfated psolusosides from *Psolus fabricii*) may enhance cytotoxicity while reducing hemolytic activity, a common limitation of these compounds [111]. Developing glycoside analogs with improved pharmacokinetics, such as the seriniquinone glycoside with enhanced solubility, represents a promising avenue [104].

Combination therapies present another frontier. The synergistic effects of glycosides with radiation or immunomodulators (e.g., CpG-ODN) warrant further exploration in preclinical models, particularly for overcoming drug resistance in solid tumors [106,108]. Additionally, leveraging microbial biotransformation (e.g., *B. licheniformis*-mediated glycosylation) could enable large-scale production of rare glycosides, addressing supply challenges for clinical trials [104].

Long-term safety and toxicity profiles must also be evaluated. While frondoside A shows minimal toxicity in mice, high-dose studies of highly sulfated glycosides (e.g., quadrangularisosides) reveal hemolytic effects that require structural modification to mitigate [108,111]. Overall, marine glycosides hold significant potential as anticancer agents, with ongoing research aimed at bridging preclinical efficacy to clinical translation. Cucumarioside A_2_-2 represents a promising candidate for developing immuno-cytotoxic therapies. Future research should focus on optimizing its pharmacokinetics—such as improving half-life through nanoliposomal encapsulation—and evaluating its efficacy in combination with PD-1/PD-L1 blockers in preclinical cancer models [25]. Additionally, clinical trials are warranted to assess its safety and antitumor activity in patients with hormone-refractory prostate cancer or other solid tumors. The compound’s ability to modulate both cancer cell survival and immune cell function highlights its potential as a novel agent in personalized cancer therapy.

### 3.6. Ilimaquinone

Ilimaquinone is a sesquiterpene quinone.


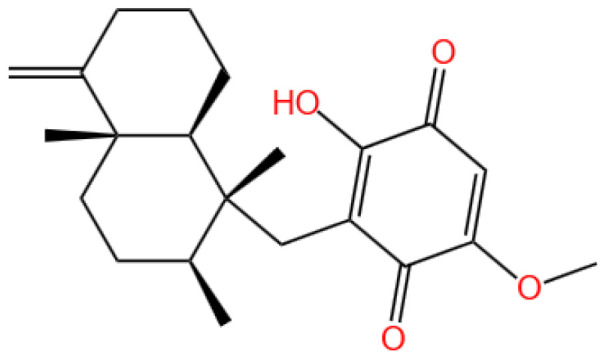


#### 3.6.1. Background and Discovery

Ilimaquinone is a bioactive sesquiterpene quinone isolated primarily from marine sponges of the genus *Hippospongia*, such as *Hippospongia metachromia* and *Dactylospongia elegans* [28,112]. First identified in the 1990s from a deep-water sponge, this compound belongs to a class of natural products characterized by a quinone moiety and a bicyclic ring structure, which underlies its diverse biological activities [113]. The compound’s discovery stemmed from screening marine natural products for cytotoxicity, and subsequent studies revealed its potential in cancer therapy and antiviral applications [114,115].

Structural analogs of ilimaquinone, such as 5-epi-ilimaquinone, have been isolated from the same sponge species, demonstrating the structural diversity within this class [28]. The compound’s unique chemical framework—including a quinone ring and specific stereochemistry—has made it a target for synthetic modification to enhance bioactivity and pharmacokinetic properties [116].

#### 3.6.2. Mechanism of Action

Ilimaquinone exerts its biological effects through multiple interconnected pathways, primarily targeting cellular stress responses, energy metabolism, and apoptotic machinery. In cancer cells, it induces apoptosis via the mitochondrial pathway, evidenced by cytochrome c release, caspase-3 activation, and mitochondrial membrane potential depolarization [28,117]. This process is often mediated by the upregulation of pro-apoptotic proteins (e.g., Bax) and downregulation of anti-apoptotic proteins (e.g., Bcl-2) [117].

The compound also activates the DNA damage response (DDR) by inducing double-strand breaks, leading to the accumulation of γ-H2AX and activation of checkpoint kinases (ATM/ATR) [29]. This DDR activation triggers cell cycle arrest at the G2/M phase and subsequent apoptotic cell death, particularly in p53-mutant colorectal cancer cells [29]. Additionally, ilimaquinone inhibits pyruvate dehydrogenase kinase 1 (PDK1), reducing aerobic glycolysis and depleting cellular ATP, which sensitizes cancer cells to apoptosis [30].

In normal cells, ilimaquinone disrupts Golgi membrane integrity and inhibits protein transport, a mechanism first observed in vitro [115]. This property may contribute to its antiproliferative effects by interfering with secretory pathway function in rapidly dividing cells. Furthermore, the compound modulates the Wnt/β-catenin pathway, suppressing neovascularization in age-related macular degeneration models by inhibiting β-catenin nuclear translocation [118].

#### 3.6.3. Experimental Evidence

Preclinical studies have validated ilimaquinone’s efficacy across various cancer types. In vitro, the compound exhibits cytotoxicity against human colorectal carcinoma cells (HCT-116) with an IC_50_ of 4.2 μM, inducing apoptosis through mitochondrial dysfunction and caspase activation [117]. It also demonstrates potent activity against melanoma cells (501Mel), reducing cell viability via reactive oxygen species (ROS)-mediated DNA damage [28].

In breast and oral squamous cell carcinoma models, ilimaquinone inhibits cell proliferation, migration, and invasion while promoting autophagy-dependent cell death [119,120]. For example, in oral cancer cells, the compound increases LC3II conversion and p62 degradation, markers of autophagic flux, while activating the AMPK/mTOR pathway [119].

Animal studies show that ilimaquinone (10 mg/kg i.p.) suppresses tumor growth in xenograft models of colorectal cancer, reducing tumor volume by 38% within two weeks without significant systemic toxicity [117]. In a mouse model of age-related macular degeneration, the compound (2 mg/kg) inhibits choroidal neovascularization by 55%, attributed to its suppression of VEGF signaling and endothelial cell migration [118].

Pharmacokinetic studies in rats reveal stereoselective metabolism of ilimaquinone epimers, with the (5R)-isomer exhibiting a longer half-life (2.3 h) and higher bioavailability than its (5S)-counterpart [116]. This finding highlights the importance of stereochemistry in optimizing the compound’s therapeutic index.

#### 3.6.4. Future Perspectives

Future research on ilimaquinone should focus on three key areas: structural optimization, combination therapy, and clinical translation. Synthetic analogs with enhanced stability and reduced toxicity, such as halogenated or methylated derivatives, may overcome the compound’s short half-life and poor water solubility [116]. For instance, modifying the quinone ring to improve antioxidant capacity could reduce off-target ROS damage in normal tissues.

Combination strategies hold promise for enhancing efficacy. Preclinical data suggest that ilimaquinone synergizes with DNA-damaging agents (e.g., cisplatin) in p53-mutant cancers, as DDR activation sensitizes cells to chemotherapy [29]. Additionally, pairing ilimaquinone with autophagy inhibitors (e.g., hydroxychloroquine) may overcome autophagy-mediated drug resistance in solid tumors [119].

Clinical trials are warranted to evaluate ilimaquinone’s safety and efficacy in humans, particularly for metastatic colorectal cancer and age-related macular degeneration. Nanoparticulate delivery systems, such as liposomes or polymeric micelles, could improve tumor targeting and reduce systemic exposure, addressing pharmacokinetic limitations [116]. Finally, exploring the compound’s antiviral potential against RNA viruses like SARS-CoV-2—based on in silico predictions of its binding to viral proteases—represents an emerging research frontier [121].

### 3.7. Aplidin (Plitidepsin or Dehydrodidemnin B)

Aplidin (plitidepsin or dehydrodidemnin B) is a cyclic depsipeptide.


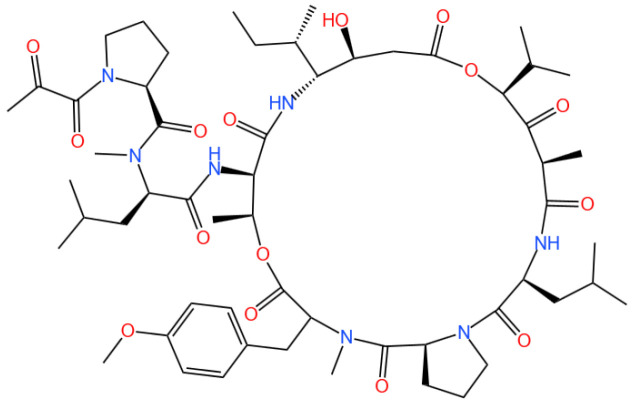


#### 3.7.1. Background and Discovery

Aplidin, also known as plitidepsin or dehydrodidemnin B, is a cyclic depsipeptide derived from the marine tunicate *Didemnum molle* and other related species. First isolated in the 1980s, this natural product belongs to a class of bioactive compounds produced by marine invertebrates, distinguished by its unique macrocyclic structure and potent biological activity [122,123]. The compound’s discovery stemmed from screening programs targeting marine natural products for anticancer potential, leading to its development by PharmaMar as a novel therapeutic agent [122].

Structurally, Aplidin features a cyclic peptide backbone with ester linkages, conferring stability and membrane permeability. Its chemical synthesis and semisynthesis have been optimized, enabling large-scale production for preclinical and clinical studies [10]. The compound’s mechanism of action was initially characterized in cancer cells, but recent research has expanded its therapeutic scope to viral infections, particularly COVID-19 [36,124].

#### 3.7.2. Mechanism of Action

Aplidin exerts its biological effects through multiple interconnected pathways, primarily targeting protein synthesis and cellular stress responses. The compound binds to eukaryotic elongation factor 1A2 (eEF1A2), a protein crucial for aminoacyl-tRNA delivery during translation elongation. This binding disrupts protein synthesis, leading to endoplasmic reticulum (ER) stress and the unfolded protein response (UPR) [31,32].

In cancer cells, ER stress induced by Aplidin activates the c-Jun *N*-terminal kinase (JNK) pathway, promoting pro-apoptotic signaling and inhibiting anti-apoptotic proteins (e.g., Bcl-2) [125,126]. Concurrently, the compound suppresses autophagy by blocking the autophagosome–lysosome fusion, leading to proteotoxic cell death [31]. This dual mechanism of ER stress induction and autophagy inhibition makes Aplidin effective against drug-resistant cancer cells [33].

As an antiviral agent, Aplidin interferes with SARS-CoV-2 replication by targeting host factors essential for viral protein synthesis. Subcellular analysis shows that the compound disrupts viral assembly by altering ER-Golgi membrane dynamics, preventing the maturation of viral particles [124]. Additionally, Aplidin modulates the host immune response, reducing pro-inflammatory cytokine release and alleviating lung injury in COVID-19 models [36,127].

#### 3.7.3. Experimental Evidence

Preclinical studies have validated Aplidin’s efficacy in multiple cancer types. In vitro, the compound exhibits cytotoxicity against myeloma cells (IC_50_ = 0.1–1 nM), inducing apoptosis and inhibiting osteoclast differentiation, which reduces bone resorption in multiple myeloma [33,34]. In vivo, Aplidin (1–5 mg/kg i.p.) suppresses tumor growth in xenograft models, with synergistic effects when combined with dexamethasone [34,35].

Clinical trials have demonstrated Aplidin’s therapeutic potential in relapsed/refractory multiple myeloma. A Phase II trial showed a 28% overall response rate in patients treated with Aplidin (0.35 mg/m^2^ every 2 weeks), with manageable side effects primarily involving gastrointestinal and hematological toxicity [35]. Pharmacokinetic studies in humans reveal a half-life of 2–4 h, with renal excretion as the primary elimination route [128].

In COVID-19 patients, a Phase III trial showed that Aplidin (0.3 mg/kg i.v.) reduced the time to clinical improvement by 3 days compared to placebo in hospitalized adults with moderate disease [36]. Mechanistic studies indicated that the compound inhibits viral replication and dampens hyperinflammation, supporting its use as an adjunct therapy [124,127].

#### 3.7.4. Future Perspectives

Future research on Aplidin should focus on three key directions: combination therapies, structural optimization, and expanded therapeutic indications. Combination with immunomodulatory agents (e.g., checkpoint inhibitors) or autophagy activators may enhance anticancer efficacy, while pairing with antiviral drugs could improve outcomes in severe viral infections [31,123].

Structural modification of Aplidin to improve pharmacokinetics—such as enhancing stability or reducing immunogenicity—represents a promising avenue. Semisynthetic platforms have enabled the production of analogs with improved solubility and half-life, which may overcome limitations of the parent compound [10].

Clinical exploration of Aplidin in other cancers (e.g., solid tumors) and viral diseases (e.g., influenza) is warranted. Preclinical data suggest activity against renal cell carcinoma and lymphoma, while its mechanism of action may extend to other RNA viruses [129,130]. Additionally, developing targeted delivery systems (e.g., liposomal formulations) could minimize systemic toxicity and enhance tumor accumulation [123].

### 3.8. Summary of the Section

Marine-derived compounds in cancer therapy exhibit unified strategies to target dysregulated cellular pathways. Microcolin H activates autophagy via PITPα/β inhibition, while nortopsentin/topsentin disrupt kinase signaling. Bryostatin modulates PKC to enhance immune cell function, and Benzosceptrin C degrades PD-L1 to restore T cell immunity. Glycosides like cucumarioside A_2_-2 combine cytotoxicity with macrophage polarization, ilimaquinone induces mitochondrial apoptosis, and Aplidin disrupts protein synthesis via eEF1A2. Despite distinct targets, these compounds converge on disrupting cancer cell survival, exemplifying marine natural products as rich sources of innovative therapeutic scaffolds.

## 4. Marine-Derived Compounds in Renal Disease Treatment

### 4.1. S14

13-hydroxyglucopiericidin A (referred to as S14) is a piericidin glycoside analog.


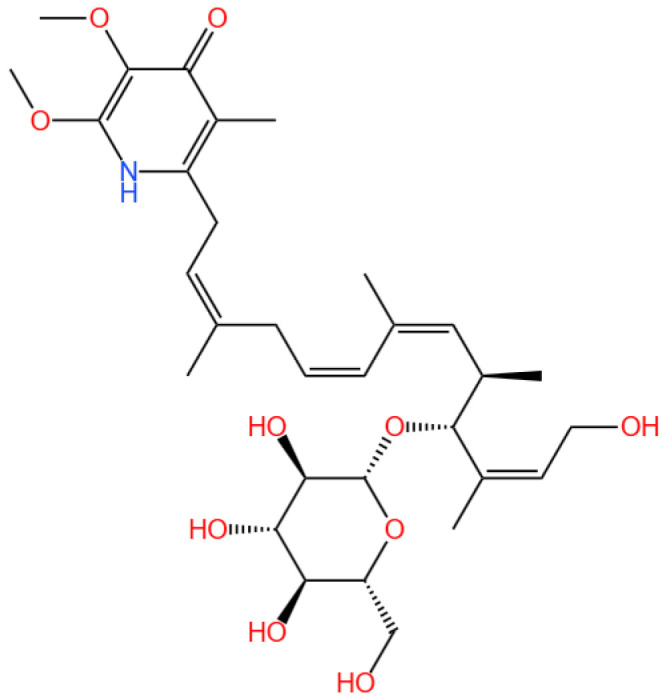


#### 4.1.1. Renal Disease Landscape

AKI is a critical clinical condition characterized by a rapid decline in renal function, often leading to chronic kidney disease or end-stage renal disease [131]. Current management strategies are largely supportive, emphasizing the urgent need for targeted therapeutic interventions. Oxidative stress, inflammation, and vascular damage are key contributors to AKI pathophysiology, with oxidative stress playing a dominant role by inducing mitochondrial dysfunction and apoptosis [132].

#### 4.1.2. The Role of S14

Previously, 43 natural piericidins from two marine-derived *Streptomyces* strains [133,134,135] have emerged as promising candidates for drug development due to their unique bioactivities. Several of these natural piericidins have demonstrated promising potential for treating renal cancer by targeting peroxiredoxin 1 (PRDX1) [136]. Among these, the piericidin glycoside analog 13-hydroxyglucopiericidin A (referred to as S14) has shown the ability to slow down renal fibrosis and AKI by enhancing autophagy and maintaining mitochondrial balance, acting as a novel activator of liver kinase B1 (LKB1) [11]. Moreover, S14 might also be effective in treating AKI through additional critical pathways. However, S14’s clinical translation is hindered by poor bioavailability, rapid metabolism, and limited renal targeting.

#### 4.1.3. Delivery System and Efficacy

**Figure 4:**
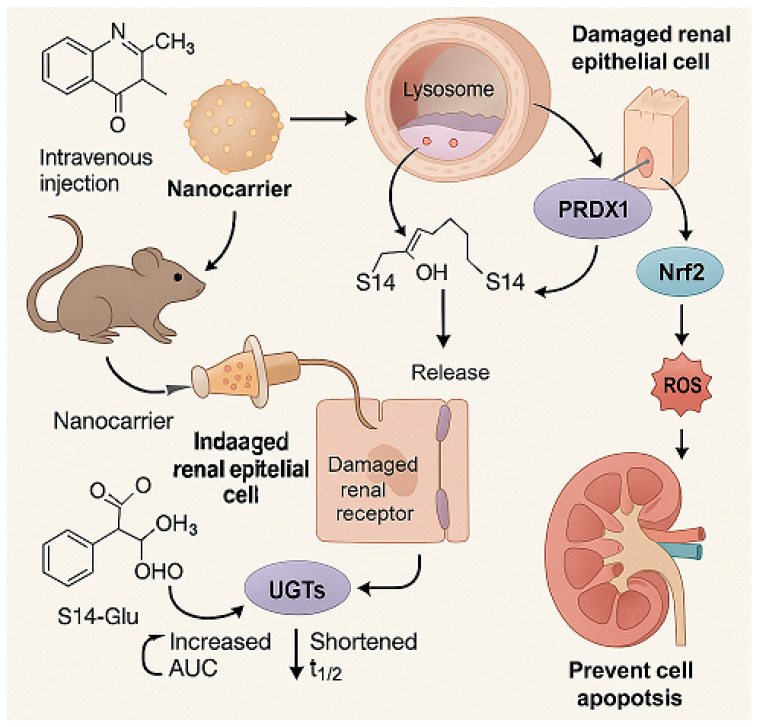
Interaction and modulation of the PRDX1/Nrf2 pathway by the S14-loaded nanodelivery system in AKI treatment. This schematic illustrates the mechanism of action for the marine-derived compound S14 in the treatment of acute kidney injury (AKI). Administered via intravenous injection into an AKI mouse model, S14 is delivered through a nanocarrier system that targets Kim-1 receptors on damaged renal cells. Upon cellular uptake, the nanocarrier releases S14 within lysosomes, where the compound interacts with peroxiredoxin 1 (PRDX1) to modulate the PRDX1/Nrf2 pathway. This modulation reduces reactive oxygen species (ROS) production, a key contributor to AKI pathogenesis. Additionally, the schematic depicts S14’s biotransformation into S14-Glu via UDP-glucuronosyltransferases (UGTs), which enhances renal distribution and pharmacokinetic properties—including an increased area under the curve (AUC) and shortened half-life (t_1/2_). These effects collectively prevent renal cell apoptosis and mitigate AKI progression.

Recently, Yu et al. (2024) developed a kidney-targeting nanodelivery system to address these challenges by investigating S14’s mechanism of action and optimizing its pharmacokinetics through an advanced delivery strategy [13]. The study employed in vitro and in vivo models to evaluate S14’s therapeutic potential, with specific emphasis on defining dosage parameters and incorporating positive controls for comparative analysis. In vitro, S14’s binding to PRDX1 was confirmed via surface plasmon resonance (SPR) and cellular thermal shift assays (CETSA), demonstrating specific interaction with the Cys83 residue to enhance peroxidase activity and promote nuclear translocation of PRDX1, thereby activating the Nrf2/HO-1/NQO1 pathway to reduce oxidative stress and apoptosis in renal tubular cells (Figure 4).

For in vivo evaluation, a unilateral ischemia-reperfusion injury (UIRI) mouse model was utilized, with treatment groups receiving either free S14 (5 mg/kg); S14-loaded nanoparticles (5 mg/kg S14 equivalent); or the positive control edaravone (30 mg/kg), a clinically used antioxidant for AKI. Renal function markers (serum creatinine and blood urea nitrogen), histopathological changes, and oxidative stress indicators (malondialdehyde and superoxide dismutase) were assessed as endpoints. The kidney-targeting nanodelivery system, composed of pH-sensitive L-serine-modified chitosan, exhibited enhanced pharmacokinetics compared to free S14, showing a 2.5-fold increase in the area under the curve (AUC) and a 1.8-fold extension in half-life. Targeting specificity was achieved via interaction with kidney injury molecule-1 (Kim-1), enabling pH-responsive drug release in renal tissues. LC-MS/MS analysis confirmed that S14-loaded nanoparticles achieved a renal concentration of 12.5 ± 1.8 μg/g at 6 h—2.8-fold higher than free S14—with plasma levels reduced by 67%. This validates renal targeting and supports mechanistic efficacy.

Notably, S14-loaded nanoparticles significantly reduced AKI-induced oxidative stress, inflammation, and apoptosis, outperforming both free S14 and the positive control edaravone at the tested dosages. Toxicity assessments in mice revealed no significant adverse effects at the administered doses (up to 20 mg/kg for nanoparticles), as confirmed by hematological and biochemical analyses. This study highlights the innovative use of a nanodelivery system to enhance the therapeutic index of a marine-derived compound, with clear dosage specifications and positive control comparisons that facilitate translational references for clinical research. Compared to previous AKI therapies using antioxidants like resveratrol (typically tested at 10–50 mg/kg), S14’s mechanism via PRDX1 and kidney-specific targeting represent notable advancements, though further dose-ranging studies and long-term toxicity evaluations are warranted to establish its clinical feasibility.

#### 4.1.4. Future Research

Future research should focus on optimizing the nanodelivery system for clinical use, including scaling up production and conducting long-term safety studies. Expanding the investigation to other renal injury models and exploring combination therapies with existing AKI treatments could further validate S14’s clinical potential. Additionally, the development of similar delivery platforms for other marine-derived compounds could broaden the scope of marine drug applications.

The study identifies S14 as a novel therapeutic agent for AKI, targeting PRDX1 to mitigate oxidative stress and renal injury. The development of a pH-sensitive, kidney-targeting nanodelivery system addresses the pharmacokinetic limitations of S14, enhancing its therapeutic efficacy. These findings underscore the potential of integrating marine-derived compounds with advanced delivery technologies for effective treatment of renal diseases.

### 4.2. HN-001

HN-001 is a bioactive compound.


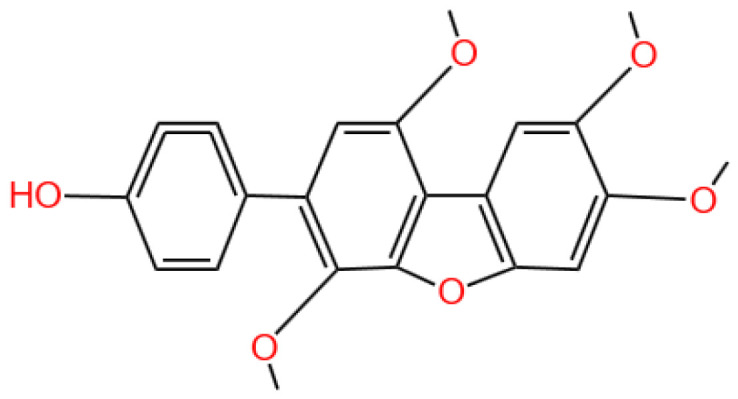


#### 4.2.1. MAFLD Prevalence and Mechanisms

**Figure 5:**
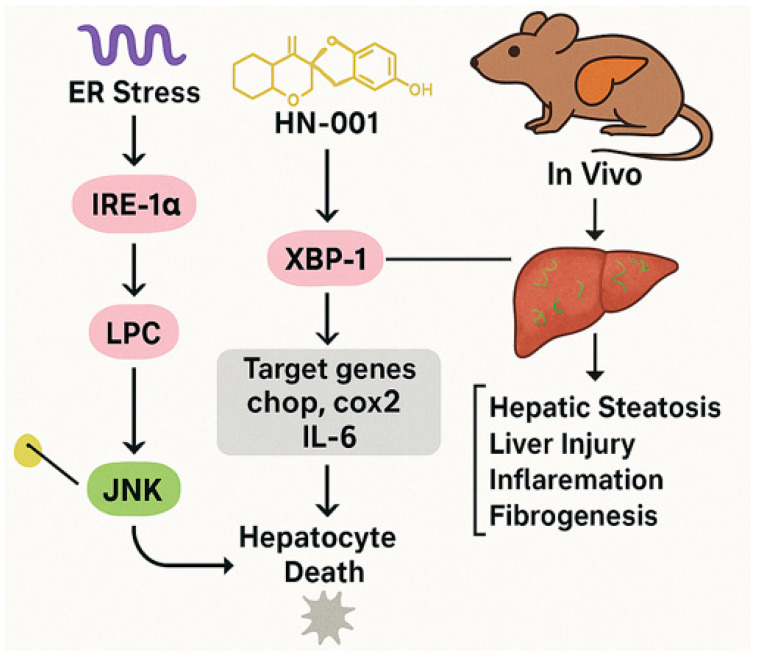
Mechanism of action of HN-001 in alleviating lipotoxicity and MAFLD. This schematic illustrates the molecular pathway by which the compound HN-001, derived from the marine fungus *Aspergillus* sp. C1, mitigates lipotoxicity and metabolic-associated fatty liver disease (MAFLD). HN-001 inhibits IRE-1α-mediated XBP-1 splicing, preventing XBP-1’s nuclear translocation and subsequent activation of target genes such as chop, cox2, and IL-6. This inhibition leads to a reduction in endoplasmic reticulum (ER) stress and hepatocyte death. Additionally, HN-001 suppresses PLA2 activity, which is associated with decreased lysophosphatidylcholine (LPC) levels and amelioration of lipotoxicity. The suppression of the pro-apoptotic JNK pathway is also depicted as part of the therapeutic action of HN-001. In vivo results show that chronic administration of HN-001 in mice alleviates hepatic steatosis, liver injury, inflammation, and fibrogenesis associated with MAFLD through modulation of the PLA2/IRE-1α/XBP-1s axis and JNK signaling.

MAFLD has become the most prevalent chronic liver condition globally, linked to ectopic lipid accumulation in the liver [72]. This condition progresses through stages ranging from simple steatosis to nonalcoholic steatohepatitis (NASH), potentially leading to cirrhosis and hepatocellular carcinoma [137,138]. Lipotoxicity, primarily driven by the ectopic accumulation of toxic lipids, plays a central role in MAFLD progression by inducing hepatocyte death through mechanisms, such as endoplasmic reticulum (ER) stress and apoptosis [139]. The IRE-1α/XBP-1s axis and c-Jun *N*-terminal kinase (JNK) signaling pathways are critical mediators of ER stress-induced apoptosis (Figure 5) [140,141].

#### 4.2.2. HN-001’s Mechanism

HN-001, derived from the marine fungus *Aspergillus* sp. C1, inhibits PLA2 enzymatic activity, reducing lysophosphatidylcholine levels and preventing JNK pathway activation (Figure 5). It also suppresses ER stress markers and pro-apoptotic signaling, effectively reversing lipotoxic effects in cells.

A natural compound known as HN-001, with a molecular weight of 380.4, has been isolated from the marine fungus *Aspergillus* sp. C1. It has been previously recognized for its antibacterial, antioxidant, anti-inflammatory, and antiausterity properties [142,143,144]. Despite these known benefits, its potential application as a treatment for MAFLD and the specific molecular mechanisms involved have not yet been investigated.

#### 4.2.3. In Vivo and In Vitro Findings

A recent study by Rao et al. (2024) underscored the critical role of lipotoxicity driven by toxic lipid accumulation in the liver, which progresses MAFLD through stages that may lead to severe conditions like cirrhosis and hepatocellular carcinoma [12]. The effects of this compound HN-001 was tested both in vitro using HepG2 cells and in vivo using a mouse model induced by a high-fat, high-cholesterol diet. Key findings reveal that HN-001 not only reverses lipotoxic effects in cells by suppressing ER stress markers and pro-apoptotic signaling but also inhibits PLA2 enzymatic activity, which reduces lysophosphatidylcholine levels and prevents JNK pathway activation. In vivo, HN-001 effectively reduced MAFLD symptoms without toxicity. The molecular mechanism involves targeting PLA2, thereby inhibiting the upstream stress pathways. Compared to other PLA2 inhibitors like Methyl arachidonyl fluorophosphonate (MAFP) [145], HN-001 shows superior efficacy, suggesting its distinct dual action on PLA2 and downstream pathways.

#### 4.2.4. Future Research Needs

Future research should focus on clinical translation, examining pharmacokinetics and safety in trials, exploring additional molecular targets, and potential combination therapies with existing anti-MAFLD agents. This study highlights the potential of marine fungi as a source of novel bioactive compounds, with HN-001 standing out as a promising therapeutic for MAFLD by targeting PLA2 and mitigating lipotoxicity.

Its marine origin underscores the potential of marine fungi as a reservoir for novel bioactive compounds.

### 4.3. Summary of the Section

Renal disease therapeutics from marine sources primarily leverage mechanisms to counteract oxidative stress, inflammation, and mitochondrial dysfunction. S14 activates PRDX1/Nrf2 signaling to reduce renal injury, while HN-001 suppresses lipotoxicity via PLA2 inhibition. Both compounds exemplify how marine-derived molecules restore cellular homeostasis by targeting stress-responsive pathways (e.g., ER stress and JNK signaling), underscoring a common strategy of mitigating injury through dual regulation of oxidative stress and inflammatory cascades. This highlights the translational potential of marine compounds in addressing the shared pathophysiology of acute and chronic renal disorders.

## 5. Marine-Derived Compounds in Atherosclerosis Treatment

### 5.1. Equisetin

Equisetin is a bioactive compound, specifically a tetramic acid analog.


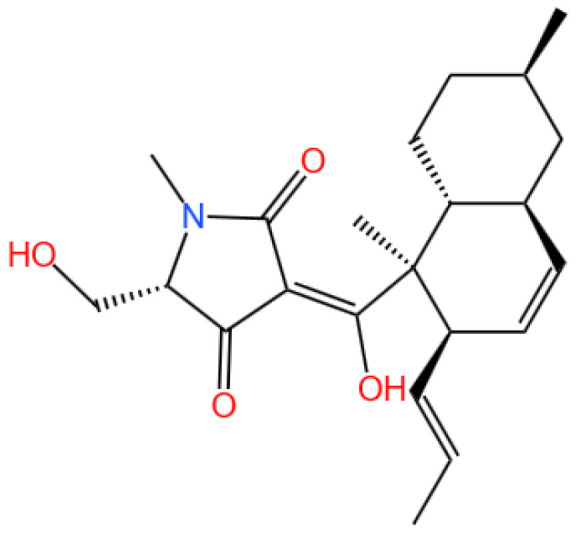


#### 5.1.1. Background and Discovery

Atherosclerosis is the leading cause of cardiovascular-related mortality worldwide, resulting in ischemic conditions such as coronary artery disease and carotid artery stenosis [146,147]. It is primarily driven by lipid accumulation and chronic inflammation within arterial walls [148]. Macrophages play a pivotal role in the disease’s progression by internalizing oxidized low-density lipoproteins (oxLDLs), transforming into foam cells, and exacerbating inflammatory responses [149]. The balance between pro- and anti-inflammatory mechanisms is critical in controlling lesion development [150].

Equisetin (EQST), isolated from the marine sponge-associated fungus *Fusarium equiseti* [37], has exhibited diverse biological activities, including antibacterial, anti-inflammatory, and lipid-lowering effects [37,151,152,153,154]. While EQST’s role in obesity and lipid metabolism has been previously studied, its potential anti-atherosclerotic effects remained unexplored until this investigation.

#### 5.1.2. Mechanism of Action

**Figure 6:**
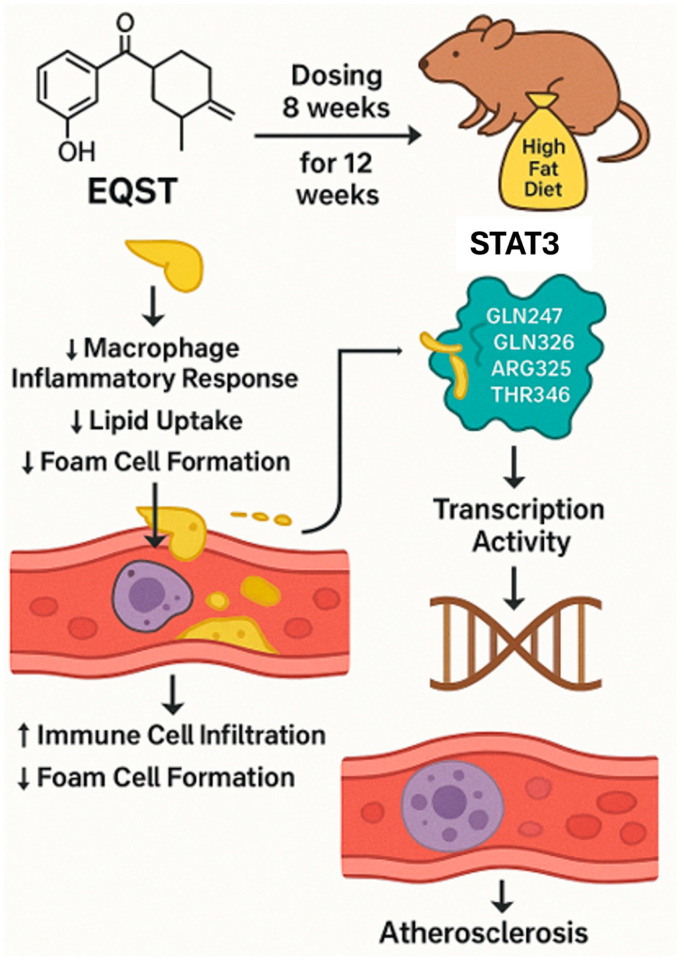
Equisetin (EQST) attenuates atherosclerosis in ApoE^−/−^ mice on a high-fat diet. This schematic illustrates the anti-atherosclerotic effects of Equisetin (EQST) in ApoE^−/−^ mice subjected to a high-fat diet (HFD). The top panel displays the chemical structure of EQST and an overview of the experimental design, indicating dosing after 8 weeks and continued for 12 weeks. The central diagram depicts a cross-section of an artery, showing how EQST inhibits the macrophage inflammatory response, lipid uptake, and foam cell formation—key processes in atherosclerosis development. Notably, EQST binds to STAT3 at specific residues (GLN247, GLN326, ARG325, and THR346), inhibiting its transcription activity, which is crucial for regulating atherosclerosis. The bottom panel highlights the outcomes of EQST treatment: reduced immune cell infiltration and foam cell formation within arterial walls lead to decreased atherosclerosis.

Recently, Yang et al. (2024) employed both in vitro and in vivo approaches. RAW264.7 macrophages and bone marrow-derived macrophages (BMDMs) were used to assess EQST’s effects on foam cell formation, lipid uptake, and inflammatory responses [38]. ApoE^−/−^ mice on an HFD were treated with EQST or rosuvastatin as a positive control. Biochemical assays, histological analyses, molecular docking, and protein interaction studies (e.g., isothermal titration calorimetry and cellular thermal shift assays) were performed to elucidate EQST’s mechanism of action, particularly its interaction with STAT3 (Figure 6).

EQST significantly inhibited foam cell formation in macrophages by reducing lipid droplet accumulation and oxLDL uptake. It downregulated the expression of scavenger receptors CD36 and SR-A at both the mRNA and protein levels. In addition, EQST suppressed macrophage migration, invasion, and the production of pro-inflammatory cytokines (IL-6, IL-1β, and TNF-α).

Mechanistically, EQST directly bound to STAT3, forming hydrophobic and hydrogen bonds, as confirmed by molecular docking and calorimetry. This interaction inhibited STAT3’s phosphorylation, nuclear translocation, and transcriptional activity. In vivo, EQST reduced the atherosclerotic plaque size and lipid accumulation in ApoE^−/−^ mice, outperforming rosuvastatin in lipid-lowering and anti-inflammatory effects.

Compared to similar studies, EQST’s dual action on lipid metabolism and inflammation positions it as a superior candidate for atherosclerosis therapy. Marine-derived compounds such as fucoxanthin and astaxanthin have shown comparable anti-inflammatory effects may comprise the STAT3-targeting specificity of EQST [155,156]. Furthermore, EQST’s efficacy surpasses that of conventional statins in reducing macrophage-driven inflammation, suggesting a distinct therapeutic mechanism.

#### 5.1.3. Future Perspectives

Future studies should focus on the pharmacokinetics and safety profile of EQST in clinical settings. Exploring its effects on glycosaminoglycan-mediated lipid deposition and its potential synergy with existing therapies could broaden its therapeutic applications. Additionally, the development of EQST analogs with enhanced bioavailability may further optimize its clinical utility.

### 5.2. Summary of the Section

Equisetin’s anti-atherosclerotic activity exemplifies marine compounds’ ability to target the intersection of lipid metabolism and inflammation. By binding STAT3 to suppress macrophage activation and lipid uptake, Equisetin mirrors mechanisms seen in other marine agents (e.g., Benzosceptrin C’s immune modulation and Microcolin H’s autophagy induction). This underscores a unifying theme: marine natural products often disrupt disease progression by modulating conserved signaling hubs (e.g., STAT3, PKC, and autophagic pathways) that bridge lipid dysregulation and inflammatory responses, offering multifaceted solutions for complex diseases like atherosclerosis.

## 6. Other Marine-Derived Compounds

While this review focuses on alkaloids, macrolides, and other small-molecule therapeutics, it is noteworthy that marine-derived polysaccharides, fatty acids, phlorotannins, and pigments have also demonstrated significant bioactivity in preclinical studies. These compounds—including fucoidans (polysaccharides) [157], omega-3 fatty acids [158], and fucoxanthin (a carotenoid) [159]—have been extensively reviewed elsewhere for their roles in immunomodulation, antioxidant effects, and metabolic regulation. Their exclusion here reflects the scope of this work, which prioritizes underexplored mechanisms and translational potential in cancer, renal, and metabolic diseases.

### 6.1. Fucoidan

Fucoidan is a sulfated polysaccharide.


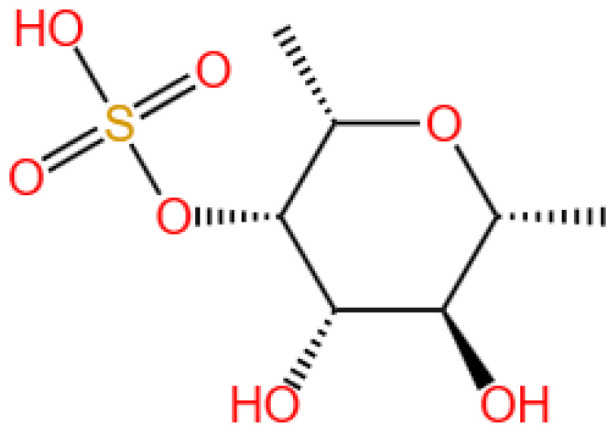


#### 6.1.1. Background and Discovery

Fucoidan is a sulfated polysaccharide predominantly isolated from brown algae (e.g., *Fucus vesiculosus* and *Sargassum* spp.) and marine invertebrates, such as sea cucumbers. Its structure comprises fucose residues with variable sulfation patterns and branching, which underlie its diverse biological activities [157,160]. First characterized in the early 20th century for its anticoagulant properties, fucoidan has gained renewed attention for its potential in cancer therapy, immunomodulation, and viral inhibition [161,162].

Marine sources yield fucoidans with distinct molecular weights and sulfation profiles. For example, fucoidan from Sargassum echinocarpum exhibits anti-breast cancer activity with a molecular weight of ~30 kDa and sulfation at C-2 and C-4 of fucose residues [39], while sea cucumber-derived fucoidan shows higher sulfation density and potent anticancer effects [163,164]. Structural variations are also influenced by extraction methods: a recent study comparing dynamic maceration (DM) and ultrasound-assisted extraction (UAE) from Arctic brown algae found that UAE increased fucoidan yield by 43.2% and reduced the molecular weight, while DM preserved higher phlorotannin content [165]. Structural variations influence bioactivity, with low-molecular-weight fucoidans (e.g., LF2, <10 kDa) demonstrating enhanced tumor penetration and reduced toxicity [166,167].

#### 6.1.2. Mechanism of Action

Fucoidan exerts therapeutic effects through multiple interconnected pathways. It activates dendritic cells and natural killer cells, promoting T cell polarization and enhancing antitumor immunity. Fucoidan from Durvillaea antarctica potentiates the efficacy of anti-PD-L1 antibodies by upregulating MHC class II expression on dendritic cells [168], while Ecklonia cava fucoidan stimulates NK cell cytotoxicity via IFN-γ secretion [21]. Fucoidan modulates the gut microbiota to enhance tryptophan metabolism, increasing the production of anti-inflammatory metabolites (e.g., indole-3-acetic acid) that suppress tumor growth [164]. It also inhibits angiogenesis by blocking VEGF receptor 2 phosphorylation [169]. The compound induces ferroptosis in HepG2 cells via ROS-mediated glutathione depletion and lipid peroxidation [170] and triggers apoptosis in colorectal cancer cells through mitochondrial membrane potential disruption [40]. Fucoidan blocks the Wnt/β-catenin pathway by binding to the Frizzled-8 receptor, reducing β-catenin nuclear translocation in breast cancer cells [171], and activates the STING-TBK1-IRF3 pathway to induce a type I interferon response in lung cancer [172].

#### 6.1.3. Experimental Evidence

Preclinical studies have validated fucoidan’s efficacy across diverse cancer models. Fucoidan inhibits the proliferation of breast (MCF-7), colorectal (HCT-116), and ovarian cancer cells with IC_50_ values of 50–200 μg/mL, often by arresting the cell cycle at the G2/M phase [39,40,41]. Notably, fucoidans extracted from Fucus vesiculosus and Fucus serratus via DM showed potent cytotoxicity against HeLa cells, with concentration-dependent cell death induction over 48 h [165]. It sensitizes drug-resistant cells to chemotherapy; for example, fucoidan-LF2 enhances oxaliplatin efficacy in pancreatic cancer by reducing M2 macrophage polarization [166]. In nude mice with UM-UC-3 bladder tumors, fucoidan (800 μg/kg i.p.) suppresses tumor growth by 40% via autophagy inhibition and CD8+ T cell recruitment [42]. A selenium-fucoidan nanoparticle formulation reduces HepG2 tumor volume by 65% in xenograft models with minimal hepatotoxicity [173].

A recent study highlighted synergistic effects when fucoidan was combined with paclitaxel: the combination induced G2/M phase arrest in HeLa cells and showed a calculated synergistic interaction, supporting its use in complex chemotherapy for cervical carcinoma [165]. A Phase II trial showed that fucoidan (0.35 mg/m^2^) combined with dexamethasone improves progression-free survival in relapsed multiple myeloma [43]. In COVID-19 patients, fucoidan reduces viral load by interfering with the SARS-CoV-2 spike protein binding to ACE2 [44]. Nanoparticulate delivery systems enhance fucoidan’s therapeutic index. For example, fucoidan-thiolated nanoparticles loaded with doxorubicin exhibit pH-sensitive drug release in tumor microenvironments, achieving 80% tumor growth inhibition in colorectal cancer models [14,174].

#### 6.1.4. Future Perspectives

Ongoing research focuses on structural optimization, such as using GH107 family endo-fucanases for enzymatic degradation of fucoidan to generate low-molecular-weight fragments with enhanced anti-TNBC activity [167], and chemical sulfation modification to improve antiviral potency against dengue virus [44]. Combination therapies are being explored, with fucoidan showing synergism with immune checkpoint inhibitors (e.g., anti-PD-1) by reversing T cell exhaustion, as demonstrated in a mouse melanoma model where the combination reduced tumor volume by 70% [175].

Translational studies may now prioritize optimizing extraction methods (e.g., DM vs. UAE) to balance yield and bioactivity, particularly for synergistic combinations with chemotherapy [165]. Translational challenges include addressing batch-to-batch variability in the fucoidan structure [160] and developing targeted delivery systems, such as fucoidan-Ce6-chloroquine hydrogels functioning as in situ vaccines to combine photodynamic therapy with autophagy inhibition for enhanced tumor immunotherapy [176]. Expanded indications are also being investigated, as preclinical data suggest fucoidan may mitigate chemotherapy-induced alopecia [177] and heal diabetic wounds [178], warranting exploration in non-oncological applications.

## 7. Pharmacokinetics of Marine-Derived Compounds

### 7.1. Background and Characteristics

Marine-derived compounds exhibit distinct pharmacokinetic profiles shaped by their unique structural features, such as high molecular weight, sulfation patterns, and lipophilicity. These properties often lead to challenges in bioavailability, distribution, and clearance, yet also present opportunities for targeted therapeutic design. For example, the cyclic depsipeptide plitidepsin (Aplidin) demonstrates a half-life of 2–4 h in humans, with renal excretion as the primary elimination route and a volume of distribution (Vd) of 1.2 L/kg, reflecting its ability to distribute widely in tissues [128]. Similarly, the marine alkaloid Lurbinectedin shows altered pharmacokinetics when co-administered with itraconazole, a CYP3A4 inhibitor, highlighting the critical role of drug–drug interactions in clinical settings [179]. Such characteristics underscore the need for systematic evaluation of marine compound pharmacokinetics to optimize their therapeutic potential.

### 7.2. Key Pharmacokinetic Parameters and Mechanisms

#### 7.2.1. Absorption and Distribution

Low-molecular-weight compounds (e.g., plitidepsin, ~800 Da) typically exhibit better systemic absorption than high-molecular-weight polysaccharides, like fucoidan. Plitidepsin binds strongly to plasma proteins (primarily albumin), which influences its distribution and tissue penetration [128]. In contrast, sulfated polysaccharides such as fucoidan show reduced membrane permeability due to their hydrophilicity, though low-molecular-weight fragments (e.g., LF2, <10 kDa) demonstrate enhanced tumor accumulation [180]. Marine-derived anthraquinones from fungi, such as altertoxins, distribute preferentially to the liver and kidneys, attributed to their lipophilic nature [181].

#### 7.2.2. Metabolism and Excretion

Hepatic metabolism via cytochrome P450 enzymes (e.g., CYP3A4) is a common pathway for marine compounds. Plitidepsin is metabolized by CYP3A4, with 60% of its 14C-labeled form excreted in urine and 30% in feces over 72 h [128]. Anthraquinones undergo hydroxylation and glucuronidation, processes that affect their half-life and toxicity [181]. The marine-derived Hsp90 inhibitor novobiocin exhibits interpatient variability in clearance, likely due to genetic differences in metabolic enzymes [182].

#### 7.2.3. Structure–Activity Relationships

Chemical modifications can significantly impact pharmacokinetics. For instance, glycosylation of the marine compound seriniquinone improves its solubility by 50-fold, addressing poor bioavailability [3]. Sulfation in polysaccharides like fucoidan enhances water solubility but reduces cell membrane permeability, while desulfation or enzymatic degradation to low-molecular-weight fragments can improve tissue distribution [6,57]. Synthetic analogs of marine alkaloids, such as Variolin derivative PM01218, show optimized pharmacokinetics with reduced clearance in preclinical models [183].

#### 7.2.4. Clinical Implications and Challenges

Clinical trials have revealed critical pharmacokinetic insights for marine-derived drugs. Lurbinectedin’s area under the curve (AUC) increases by 38% when co-administered with itraconazole, necessitating dosage adjustments in patients taking CYP3A4 inhibitors [179]. Similarly, the marine-derived anticancer agent Trabectedin exhibits poor oral bioavailability, requiring intravenous administration and careful monitoring of hepatic function [184]. Nanoparticulate delivery systems, such as fucoidan-encapsulated selenium nanoparticles, have shown promise in extending the half-life and enhancing tumor retention, addressing limitations in systemic clearance [185].

### 7.3. Future Perspectives

Future perspectives in the pharmacokinetics of marine-derived compounds will likely focus on integrating advanced delivery technologies, computational modeling, and personalized medicine approaches to overcome current translational hurdles. First, the development of targeted nanocarriers, such as liposomes or hydrogels, will enhance tumor accumulation while minimizing systemic toxicity, addressing the poor bioavailability and short half-life often seen in marine compounds like plitidepsin. Additionally, leveraging machine learning and in silico modeling will enable more accurate prediction of absorption, distribution, metabolism, and excretion (ADME) properties, accelerating the optimization of lead compounds and reducing the need for extensive preclinical trials. Simultaneously, pharmacogenomic studies will play a critical role in tailoring dosing strategies to individual patients, particularly for compounds metabolized by enzymes like CYP3A4, as observed in Lurbinectedin trials. Structural modification strategies, such as glycosylation or sulfation adjustments, will continue to improve solubility and membrane permeability, while combinatorial therapies pairing marine-derived agents with conventional drugs or immune checkpoint inhibitors may enhance therapeutic efficacy and overcome drug resistance. Ultimately, these efforts will bridge the gap between marine natural product discovery and clinical application, unlocking the full potential of these compounds in modern therapeutics.

## 8. Conclusions

Collectively, marine-derived compounds address diverse diseases through conserved mechanisms: autophagy regulation (Microcolin H and S14), kinase inhibition (nortopsentin and Equisetin), immune checkpoint modulation (Benzosceptrin C), and metabolic balance restoration (HN-001). Their structural diversity targets shared pathological drivers—oxidative stress, inflammation, and dysregulated cell survival—across oncology, nephrology, and metabolism. However, this review also acknowledges critical limitations: ① the majority of evidence remains preclinical, with limited Phase II/III clinical data for most compounds; ② the pharmacokinetic challenges, including low bioavailability (e.g., short half-life of bryostatin-1) and poor tissue targeting; and ③ the need for multi-target mechanistic studies in complex diseases like MAFLD, where disease heterogeneity complicates single-agent efficacy.

Key future directions include optimizing nanodelivery systems to enhance tumor accumulation (e.g., S14-loaded nanoparticles); developing combinatorial therapies to overcome resistance (e.g., fucoidan-paclitaxel synergies); and advancing pharmacokinetic profiles via semisynthesis (e.g., Aplidin analogs). These efforts reinforce marine natural products as pivotal resources for tackling complex human diseases by modulating intersecting molecular pathways while underscoring the urgency of translational research to bridge preclinical promise and clinical application.

## Figures and Tables

**Table 1:** Preclinical or clinical study of marine drugs in cancer, renal, and metabolic disease therapeutics.

No.	Disease	Drug Name	Species	Dose, Route, Time	MOA	Model Inducers	Outcomes/Biological Effects	References
1	Cancer	Microcolin H	*Moorea producens*	0.1–0.5 nM (in vitro); 1–10 mg/kg i.p.	Targets PITPα/β, inhibits proliferation, induces autophagy	N/A	Suppressed tumor growth, low toxicity	[7]
2	Cancer	Nortopsentin	*Topsentia sponge*	Submicromolar (in vitro)	Inhibits CK1/GSK-3β, disrupts Wnt/β-catenin pathway	N/A	Cytotoxic to cancer cells, induces cell cycle arrest	[15,16]
3	Cancer	Topsentin	*Spongosorites sponge*		Targets CDK1/tubulin, disrupts mitosis		Active against breast cancer, inhibits biofilm	[17,18]
4	Cancer	Bryostatin	*Bugula neritina*	Clinically tested (various doses)	Activates PKC, enhances immune cell function, modulates autophagy	N/A	Reduced tumor growth, improved immune response	[19,20,21,22,23]
5	Cancer	Benzosceptrin C	*Agelas dendromorpha*	10–20 μM (in vitro); 5–10 mg/kg i.p.	Induces PD-L1 degradation via DHHC3, enhances T cell activity	N/A	Suppressed tumor growth, increased CD8+ T cells	[8,24]
6	Cancer	Cucumarioside A_2_-2	*Cucumaria japonica*	1.2–2.8 μM (in vitro); 5 mg/kg i.p.	Induces apoptosis, activates macrophages via TLR4/NF-κB	N/A	Reduced tumor volume, enhanced M1 macrophage infiltration	[25,26,27]
7	Cancer	Ilimaquinone	*Hippospongia* spp.	4.2 μM (in vitro); 10 mg/kg i.p.	Induces mitochondrial apoptosis, activates DDR, inhibits PDK1	N/A	Suppressed tumor growth, induced DNA damage	[28,29,30]
8	Cancer	Aplidin	*Didemnum molle*	0.1–1 nM (in vitro); 1–5 mg/kg i.p.	Binds eEF1A2, disrupts protein synthesis, induces ER stress	N/A	Inhibited myeloma cell growth, reduced tumor size	[31,32,33,34,35]
9	COVID-19	Aplidin	*Didemnum molle*	0.3 mg/kg i.v. (clinical)	Disrupts viral replication, modulates immune response	SARS-CoV-2	Shortened clinical improvement time in patients	[36]
10	Renal	S14	*Streptomyces* spp.	5 mg/kg (free); 5 mg/kg (nanoparticles) i.v.	Activates PRDX1/Nrf2, reduces oxidative stress, enhances autophagy	UIRI mouse model	Improved renal function, reduced injury markers	[13]
11	MAFLD	HN-001	*Aspergillus* sp. C1	Not specified (in vivo/model)	Inhibits PLA2, reduces LPC, suppresses JNK/ER stress pathway	High-fat diet	Alleviated hepatic steatosis, reduced inflammation	[12]
12	Atherosclerosis	Equisetin	*Fusarium equiseti*	Not specified (in vivo model)	Binds STAT3, inhibits its activation, reduces lipid uptake	ApoE^−/−^ HFD mouse	Reduced plaque size, decreased inflammatory markers	[37,38]
13	Cancer	Fucoidan	Brown algae/sea cucumbers	50–200 μg/mL (in vitro); 800 μg/kg i.p.	Activates immune cells, induces apoptosis, inhibits angiogenesis	N/A	Suppressed tumor growth, enhanced immune response	[39,40,41,42]
14	COVID-19	Fucoidan	Brown algae/sea cucumbers	Not specified (clinical)	Interferes with viral entry, reduces inflammation	SARS-CoV-2	Reduced viral load in patients	[43,44]

Note: No., number; MOA, mechanism of action; PITPα/β, phosphatidylinositol transfer protein α/β; CK1, casein kinase 1; GSK-3β, glycogen synthase kinase 3β; CDK1, cyclin-dependent kinase 1; PKC, protein kinase C; PD-L1, programmed death ligand-1; TLR4, toll-like receptor 4; NF-κB, nuclear factor kappa B; DDR, DNA damage response; 8. eEF1A2, eukaryotic elongation factor 1 alpha 2; PRDX1, peroxiredoxin 1; Nrf2, nuclear factor erythroid 2-related factor 2; PLA2, phospholipase A2; JNK, c-Jun N-terminal kinase; STAT3, signal transducer and activator of transcription 3.

## Data Availability

No new data were created or analyzed in this study.

## Abbreviations

The following abbreviations are used in this manuscript:

AKI	Acute kidney injury
MAFLD	Metabolic-associated fatty liver disease
NASH	Nonalcoholic steatohepatitis
DDR	DNA damage response
PRDX1	Peroxiredoxin 1
Kim-1	Kidney injury molecule-1
PD-1	Programmed cell death-1
PD-L1	Programmed cell death ligand-1
CTLA4	Cytotoxic T-lymphocyte-associated protein 4
CAR T cells	Chimeric antigen receptor T cells
Tregs	Regulatory T cells
MDSCs	Myeloid-derived suppressor cells
TNBC	Triple-negative breast cancer
PITPα/β	Phosphatidylinositol transfer protein alpha/beta isoform
LKB1	Liver kinase B1
Nrf2	Nuclear factor erythroid 2-related factor 2
HO-1	Heme oxygenase-1
NQO1	NAD(P)H quinone dehydrogenase 1
ROS	Reactive oxygen species
IRE-1α	Inositol-requiring enzyme 1 alpha
XBP-1s	Spliced X-box binding protein 1
JNK	c-Jun *N*-terminal kinase
STAT3	Signal transducer and activator of transcription 3
PLA2	Phospholipase A2
CK1	Casein kinase 1
GSK-3β	Glycogen synthase kinase-3 beta
CDK1/2/4/6	Cyclin-dependent kinase 1/2/4/6
PKC	Protein kinase C
SPR	Surface plasmon resonance
CETSA	Cellular thermal shift assay
UIRI	Unilateral ischemia-reperfusion injury
AUC	Area under the curve
LC-MS/MS	Liquid chromatography–tandem mass spectrometry
HFD	High-fat diet
oxLDL	Oxidized low-density lipoprotein
BMDMs	Bone marrow-derived macrophages
EQST	Equisetin
S14	13-hydroxyglucopiericidin A
HN-001	A compound derived from Aspergillus sp. C1
BC	Benzosceptrin C
HCQ	Hydroxychloroquine
PTX	Paclitaxel
